# Evaluation of a community-based, family focused healthy weights initiative using the RE-AIM framework

**DOI:** 10.1186/s12966-017-0638-0

**Published:** 2018-01-26

**Authors:** Mary E. Jung, Jessica E. Bourne, Heather L. Gainforth

**Affiliations:** 1 School of Health and Exercise Sciences, Faculty of Health and Social Development, University of British Columbia, Kelowna, Canada; 2 Centre for Exercise, Nutrition and health Sciences, School for Policy Studies, University of Bristol, Bristol, UK

**Keywords:** Program evaluation, Children, Healthy weight, Community, Evaluation of a community-based, Family focused healthy weights initiative using the RE-AIM framework

## Abstract

**Background:**

Childhood overweight and obesity is a major public health concern. Community-based interventions have the potential to reach caregivers and children. However, the overall health impact of these programs is rarely comprehensively assessed. This study evaluated a physical activity and healthy eating family program (Healthy Together; HT) using the RE-AIM framework.

**Methods:**

Ten sites implemented the 5-week program. Thirty-nine staff members and 277 program participants (126 caregivers [*M*_*age*_ = 35.6] and 151 children [*M*_*age*_ = 13]) participated in the evaluation. Each RE-AIM dimension was assessed independently using a mixed-methods approach. Sources of data included archival records, interviews and surveys. Effectiveness outcome variables were assessed at pre- and post-intervention and 6-month follow-up.

**Results:**

*Reach:* HT participants were almost entirely recruited from existing programs within sites. *Effectiveness:* Caregivers’ nutrition related efficacy beliefs increased following HT (*ps* < .03). Participation in HT was not associated with significant changes in physical activity or nutrition behaviour or perceived social support (*ps* > .05). Knowledge surrounding healthy diets and physical activity increased in children and caregivers (*ps* < .05). *Adoption:* Thirty-five percent of sites approached to implement HT expressed interest. The 10 sites selected recruited existing staff members to implement HT. *Implementation:* Program objectives were met 72.8% of the time and 71 adaptations were made. HT was finance- and time-dependent. *Maintenance:* Two sites fully implemented HT in the follow-up year and 5 sites incorporated aspects of HT into other programs.

**Conclusions:**

Working alongside organizations that develop community programs to conduct **c**omprehensive, arms-length evaluations can systematically highlight areas of success and challenges. Overall HT represents a feasible community-based intervention; however further support is required in order to ensure the program is effective at positively targeting the desired outcomes. As a result of this evaluation, modifications are currently being implemented to HT.

**Electronic supplementary material:**

The online version of this article (10.1186/s12966-017-0638-0) contains supplementary material, which is available to authorized users.

## Background

Childhood overweight and obesity are related to numerous proximal and distal health problems including heart disease, high blood pressure and diabetes [1–4], with evidence suggesting that children who are obese are more likely to be obese in adulthood [5]. One in every four Canadian children is considered overweight or obese [6]. Given the economic burden of obesity in Canada is estimated to be $7.1 billion annually [7], childhood overweight and obesity is a public health issue that requires urgent attention.

Several social and environmental factors have been associated with an increased risk of childhood obesity including being of aboriginal or multi-ethnic descent [8], low-socioeconomic status [9] and residing in rural and remote locations [10]. Given that children have little control over the social and environmental situations in which they live, the community has been highlighted as an important context through which to promote obesity prevention initiatives [11, 12]. Community based initiatives may be beneficial over school initiatives as they can include parents, engagement of whom is considered key in tackling pediatric obesity [13, 14]. Results from community-based interventions show potential effectiveness, with positive changes reported in clinical and behavioural outcomes at the individual level [15, 16]. While effectiveness is critical, few community-based intervention studies have examined intervention effectiveness while simultaneously assessing external indicators of success including program implementation, sustainability and maintenance within the desired context. Examination of both internal and external indicators of success are vital for assessing the long-term public health impact of an intervention.

The RE-AIM framework provides a systematic method through which to assess the overall impact of an intervention taking into consideration both internal and external validity [17, 18]. The framework outlines five dimensions to be asessed (Reach, Effectiveness, Adoption, Implementation and Maintenance) and has been used to design, implement and evaluate health promotion initiatives extensively [19–21]. Reporting on each of these dimensions enables health professionals to compare findings across interventions and establish the receptivity and sustainability of a program, enabling informed decisions about future public health initiatives. Comprehensive evaluation is also essential to establish the degree of impact that community designed and implemented programs have on the targeted population and community. Finally, it enables evaluators to determine how and why an intervention works, permitting future refinement.

The purpose of the current study was to use the RE-AIM framework to comprehensively evaluate the first iteration of Healthy Together, an education program designed for children and their caregivers, developed by a community organization.

## Methods

### Program overview

Healthy Together (HT) is a family centered education program developed by The Bridge Youth and Family Services (hereon in referred to as ‘The Bridge’) to promote healthy weights in children from vulnerable populations (i.e., rural, remote, northern, Aboriginal and multicultural communities across Canada; see http://healthy-together.ca). The first iteration of HT comprised of 3 age-based modules: Happy Healthy Beginnings (0-6 years; Module 1), Fun Healthy Habits (7-12 years; Module 2), My Life, My World, My Choice (13-18 years; Module 3). Each module consisted of five weekly sessions lasting approximately 2.5 hours, incorporating children and caregivers. Each session was designed to provide attendees with information, skills and experiences to support families in making healthy food and activity choices, and included 15–30 minutes of play-based physical activity, 30–60 minutes of group discussion and 45–60 minutes of cooking and eating together. Caregivers and children were provided with take home sheets after each session to complement discussions. Implementation sites were encouraged to adapt the discussions and handout material based on the needs of their population. Ten organizations from five regions across Canada implemented HT over 2 years (five sites per year) and took part in the program evaluation between October 2013 and June 2015.

### Participants

All caregivers and children (7–18 years) who registered for HT were eligible to take part in the evaluation of program effectiveness. Children aged 0–6 were not asked to complete any evaluation documentation. The community site director, HT program coordinator, and facilitators were all invited to participate in the evaluation. Facilitators conducted each of the HT sessions; the HT program coordinator oversaw all logistical aspects of running the program and the community director oversaw all financial and community programing. Each site had a trained onsite evaluator who obtained written consent from all participants and assent from caregivers of children 7–16 years of age.

### Design

Each RE-AIM dimension was assessed independently using a mixed-methods approach to provide an insightful evaluation enabling greater validity of inferences [22]. A mixed-methods approach was used because qualitative data can provide further and richer insights into quantitative findings. In particular, we primarily used quantitative measures to examine outcomes and qualitative findings to examine processes. Table 1 provides an overview of the variables assessed under each RE-AIM component, the data source used to assess each variable, and the data collection timeline.

**Table 1:** RE-AIM measures and data sources used to obtain information

Assessment level	Measures	Data sources	Timeline
Reach	• Eligibility criteria	• Interview data (Director) • Pre Survey items (Coordinator)	Pre-intervention
	• # Children/families in area, served by community center that meet that criteria	• Statistics Canada • Interview data (Director) • Survey data (Coordinator)	Pre-intervention
	• # Children/families registered for the program	• Summary Forms (Coordinator)	Post-intervention
	• # Exclusions	• Survey data (Coordinator)	Post-intervention
	• Recruitment strategies	• Interview data (Director) • Survey data (Coordinator)	Pre-intervention
	• Identification of facilitators and barriers to recruitment	• Interview data (Director) • Survey data (Coordinator)	Pre-intervention
Effectiveness	• Short-term attrition rates	• Survey (Caregivers, Children)	Baseline, post-intervention
	• Healthy eating and physical activity efficacy beliefs • Knowledge related to healthy eating, physical activity and screen time guidelines. • Perceived social support for physical activity and healthy eating • Healthy eating, physical activity and screen time behavour • Children’s health related quality of life	• Survey (Caregivers, Children)	Baseline, post-intervention
	• Perception of impact/consequences (positive or negative)	• Interview data (Facilitators and Directors)	Post-intervention
Adoption			
*Setting Level*	• Criteria for implementing HT	• Documentation (The Bridge)	Post-intervention
	• # of settings approached to implement		
	• # of settings that expressed interest in implementing HT		
	• Num. of sites expressing interest that were excluded		
	• Difference in settings between 1) those that expressed interest vs. no-interest. 2) exclusion vs. inclusion		
*Staff Level*	• Exclusion criteria • # staff approached/applied to be part of HT • Degree to which staff participating in HT are representative of staff at the centre	• Survey data (Directors)	Pre-intervention
	• Barriers to staff participation	• Survey data (Facilitators, Directors, Coordinators)	Pre-intervention
Implementation	• Degree to which project objectives were met	• Observations	During-Intervention
	• Degree to which program activities were administered		
	• # and type of adaptations made	• After-session survey (Facilitators)	During-intervention
	• Program attendance rate	• Observations	During-intervention
	• Barriers and facilitators to implementation	• Interview (Facilitators)	Post-intervention
	• Time cost of the intervention	• After-session surveys (Facilitators)	During-intervention
	• Financial costs of the intervention	• Documentation (The Bridge)	Post-Intervention
Maintenance			
*Individual Level*	• Long term study attrition	• Survey (Caregivers, Children)	Pre, 6-month follow-up
	• Healthy eating and physical activity efficacy beliefs • Knowledge related to healthy eating, physical activity and screen time guidelines. • Perceived social support for physical activity and healthy eating • Healthy eating, physical activity and screen time behaviour • Children’s health related quality of life	• Survey (Caregivers, Children)	Pre, 6-month follow-up
*Setting Level*	• # of sites running Healthy Together	• Surveys (Directors, Coordinators)	1-year follow-up survey and email
	• Adaptations of the program – retained elements		
	• Reasons for lack of implementation		

### Procedure

Ethical approval for this study was granted by the University of British Columbia research ethics board and the Public Health Agency of Canada ethics committee. Prior to implementing HT all facilitators received 2-days of program training from The Bridge. Training consisted of a) project orientation (including overviews of the Bridge organization, budget information and the role of evaluation), b) education on group facilitation, c) review of the program content and the facilitator manual and d) planning and role playing one program session in groups of three. Facilitators were also educated on dealing with shame and trauma and general considerations when working with children-in-care. Simultaneously, the evaluation team provided 2-day training for staff members of the implementation sites who would be acting as onsite evaluators. Onsite evaluators were responsible for collecting all survey data from participants and conducting observations.

### Measures

#### Staff surveys

Staff surveys were used to gather demographic information and information pertaining to Reach, Adoption and Maintenance using a variety of open-ended, categorical and likert-type questions. **Directors** completed surveys at baseline (*n* = 9) and 1-year follow-up (*n* = 5), while **coordinators** completed surveys at baseline (*n* = 8), post intervention (*n* = 6) and at 1-year follow-up (*n* = 4). Each survey took approximately 20-minutes to complete (Additional file 1 provides information on the data source used to assess each of the RE-AIM dimensions and the time of data collection). Facilitators completed surveys at baseline (*n* = 24) and after each program session, which took approximately 10-minutes to complete.

#### Child and caregiver surveys

Surveys were developed by the researchers to map onto the components of the HT program manual, and were administered to children and caregivers at pre-, post-, and 6-month follow-up. Demographic information was collected at baseline. The child survey measured knowledge, efficacy beliefs and behaviour in relation to healthy eating, physical activity and screen time. Health related quality of life and perceived social support for healthy eating and physical activity were also measured. Cronbach’s alpha for composite measures within the child survey ranged from .71 to .93 (see Additional file 2). All surveys were pilot tested with children (*n* = 12) to assess readability and receptiveness. The caregiver survey assessed knowledge, efficacy beliefs and behaviour in relation to health eating, physical activity and screen time. Perceived social support provided to children and caregivers’ reports of child health related quality of life was also assessed. Cronbach’s alpha for composite measures within the caregiver survey ranged from .57 to .84 (see Additional file 3). Where possible previously validated measures were used. Where no vailidated measures were available study specific measures were created and reviewed by all authors for content validity.

#### Interviews

Directors completed individual interviews at baseline (*n =* 9) and post intervention (*n* = 10). Baseline interviews including questions pertaining to HT reach and staff level adoption, while post interviews included questions pertaining to the consequences of running the HT (effectiveness). Two in-depth group interviews were conducted with facilitators post intervention (*n* = 19). One group interview was conducted in year 1 and the second in year 2. Questions assessed the dimensions of program effectiveness and implementation and lasted 1-h.

#### Observations

Onsite evaluators observed all program sessions within their community site. Evaluators rated whether facilitators met session objectives (as laid out in the program manual), presented and ran each activity, and whether facilitators adapted or added any material to the session using a ‘Yes’ or ‘No’ response scale.

#### Documentation

Internal program documents describing the process of community site involvement in HT was obtained from the Bridge to assess setting level adoption as well as financial information pertaining to the implementation of the program. Summary forms completed by each community site were obtained to assess program reach. Archival records included census data from Statistics Canada and school statistics. To gain an understanding of program maintenance, emails were sent to directors and coordinators of sites that did not return their 1-year follow-up survey.

### Data analysis

Child and caregiver survey data was analyzed using SPSS (v24, 2016). Descriptive statistics were calculated for all study variables. Data was screened for outliers using box-plots and assumptions of normality were assessed using the Shapiro-Wilk test of normality using difference scores. Partial missing data (i.e., less than 50% of a scale) were replaced using a series mean [23]. If difference scores were not normally distributed, non-parametric Wilcoxon sign-test was conducted instead of a paired-sample t-test. Independent sample t-tests and chi-squared analyses were conducted to examine differences in demographics and outcome variables between participants that dropped out of the program and those that remained at post and at 6-month follow-up. Effectiveness and individual level maintenance were analyzed using a series of paired-samples t-tests, McNemar chi-squared or Wilcoxin sign-tests. Effect sizes were calculated using odds ratios for McNemars chi-squared, *r* for Wilcoxon sign-test (0.1, 0.3, 0.5 represent a small, medium and large effect respectively; [24]) and cohen’s d for paired sample t-tests (0.2, 0.5, 0.8 represent small, medium and large effect respectively; [24]). Data were analyzed collectively for all implementation sites and where possible individually by site (i.e., sample size permitting). See Additional file 4 for a list of sites for which individual site analyses was conducted for caregivers and children. Significance was set a *p* ≤ .05.

To provide a further insights into processes that may have influenced quantitative findings, interviews and qualitative survey data were deductively analyzed using the RE-AIM framework [25]. All interviews were transcribed verbatim. Qualitative survey data was entered into an excel sheet. Two coders independantly performed a content analysis on both sets of data using the domains of RE-AIM. The coders familiarized themselves with the data by carefully reading the transcripts and survey responses. They deductively coded the data using the criteria for each of the five RE-AIM dimensions (Table 1). Any discrepancies were resolved through discussion. Coded data was then reviewed by the research team to extract illustrative quotations that provided further insight into quantitative findings.

## Results

### Reach

Two of the 10-implementation sites reported specific inclusion criteria, beyond the age requirements specified by the modules (see Additional file 5 for data used to determine program reach). At one site participants were required to be refugees or new immigrants to Canada, while at the other children had to be attending a specific school. Despite no other explicit eligibility criteria all sites targeted HT recruitment at specific populations including families of children at risk, families of Aboriginal descent, women and new immigrants, and refugees to Canada. Based on the specified eligibility criteria it was estimated that approximately 73,368 children were eligible to participate in HT across the 8 sites. Reasonable estimates of the potential eligible population were not available for two sites. A total of 223 caregivers and 398 children registered for HT at 10 sites. At the 8 sites with population estimates available 330 children registered, suggesting that the program reached approximately .45% of the potential target population. Fifty six caregivers and children were excluded from two sites for module 1 due to lack of space. One family was excluded from the program as they did not drive and the organization was unable to provide transportation. Of those that registered for the program 190 individuals self-identified as Aboriginal (31% of all registrants), 56 as immigrants (9%) and 42 children were classified as in care of the government (11% of child registrants).

#### Recruitment

The primary recruitment strategies were verbal presentations and communication with a) existing programs offered at the site, b) community partners and c) local schools and school boards. Three sites created HT information pamphlets and offered information sessions with incentives to encourage attendance. Four sites worked with local schools to recruit and offered HT as part of the school curriculum or during school time. For module 1 and 2 the majority of participants were recruited from other programs being offered at the sites.

The most commonly cited barrier to recruitment was a lack of connection to the population of interest, particularly the 13–18 year olds and a desire from this age group to participate independently of caregivers. As such, five sites ran module 3 without caregivers. A lack of trust regarding new programs with also highlighted as a barrier to recruitment. Directors explained that families with children of Aboriginal descent need extensive information about a program prior to participation to understand the motivation of the program and potential consequences of participation. Two sites stated that transportation was an issue for recruitment and that the cost of providing transportation for participants was limiting.

The most commonly cited enabler to recruitment was the presence of pre-existing programs within the site from which to invite individuals to participate. In addition, working closely with community partners helped to build the trust of participants and to recruit populations that the sites did not previously serve.

### Effectiveness

#### Study attrition

**Fig. 1:**
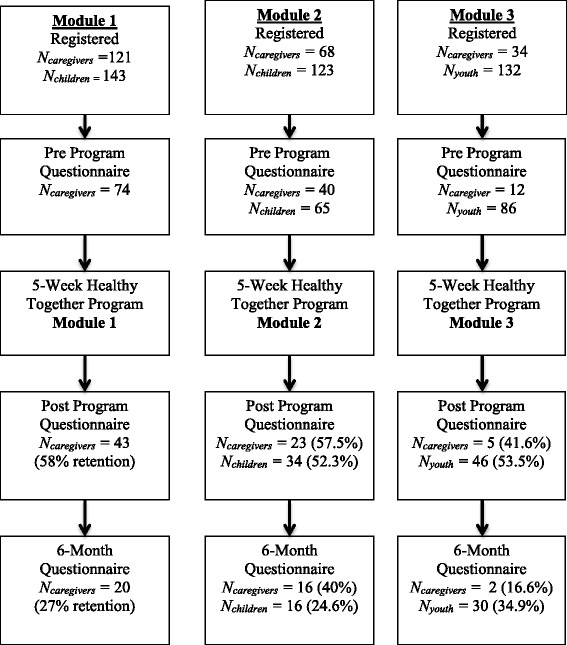
Flow of program participants through the evaluation. Percent represents participant retention in the evaluation

A total of 126 caregivers completed the baseline survey (see Fig. 1 for flow of participants through the evaluation and Additional file 6 for caregiver demographic statistics). Of these caregivers 71 completed the post survey (43.65% attrition) and 38 completed 6-month follow-up (69.84% attrition). There were no differences in age, sex, ethnicity of adult or child or marital status between participants who completed the post survey and those that did not (*ps* > .05). There was a significant difference in education level (χ^2^(5) *=* 12.12*, p* = .03) such that caregivers who completed the post survey had a higher level of education than those that did not. There were no significant differences in outcome variables pre-intervention between those that completed the post survey and those that did not (*ps* > .05).

At 6-month follow-up there was no difference in age, ethnicity of adult or child, marital status or level of education. There was a significant difference in sex between those that completed the survey and those that did not, χ^2^(1) *=* 4.13*, p* = .04. Females were 3.56 times more likely to complete the 6-month follow up than males. There were no significant differences in outcome variables pre-intervention between those that completed the 6-month follow-up survey and those that did not (*ps* > .05).

A total of 151 children completed the baseline survey (*M*_*age*_ = 13(±3); 57% female). Of these, 80 completed the post survey (47% attrition) and 46 completed 6-month follow-up (70% attrition). No differences in sex, age or any outcome variables were found between those that completed the post survey and those that did not, or those that completed the 6-month follow-up survey and those that did not (*ps* > .05).

#### Caregiver outcomes

##### Knowledge

There were no changes in knowledge of physical activity requirements for adults or children, fruit and vegetable consumption requirements for adult and children, or knowledge of screen time limits from pre to post-program, or from pre to 6-month follow-up (*ps* > .05; see Table 2 for pre, post and 6-month follow-up changes for each outcome variable). No differences in knowledge were found within individual sites (*ps* > .05) (Table 2).

**Table 2:** Changes in outcome variables for caregivers

Measure	Pre-Intervention		Post-Intervention					6-month follow-up				
	*M*	SD	*M*	SD	*n*	*p*	*es*	*M*	SD	*n*	*p*	*es*
Knowledge^a^												
Adult daily fruit and vegetable servings	9.5		17.6		65	.15	.52	16.2		35	1.0	.16
Child daily fruit and vegetables servings	20.8		42.9		25	.18	2.22	47.1		14	.22	.42
Sugary drinks	78.4		74.3		67	.58	.11	91.7		34	.69	.29
Adult minutes of PA per day	44.3		36.3		67	.15	.29	48.6		36	1.0	.25
Child minutes of PA per day	41.7		67.6		25	.13	.08	58.8		15	1.0	.25
Adult days of PA per week	50		50.7		68	.29	.21	45.9		36	.39	.24
Child days of PA per week	46.6		49.3		67	.83	.24	56.8		36	.82	1
Child screen time limits	26.7		37.7		68	.50	.20	41.7		35	.77	.31
Efficacy Beliefs												
Adult healthy eating efficacy (1–5)	2.65	1.06	3.07	0.91	65	.02*	.32	3.10	.93	32	.03*	.47
Cooking efficacy (0–100)	82.15	19.37	88.44	14.52	64	.001*	.43	88.57	12.30	32	.01*	.49
Efficacy to include child in cooking (1–5)	3.35	1.05	3.66	.90	62	.05*	.32	3.45	.98	30	.50	.12
Adult physical activity efficacy	55.36	24.64	57.87	16.13	42	.10	.26	55.15	18.11	20	.44	.18
Behaviour												
Eat breakfast^b^ (0–6)	4.00		4.00		61	.68	−.05	4.00		32	.89	.02
Caregiver fruit and vegetable consumption^b^ (0–6)	3.50		4.00		60	.57	.07	4.00		31	.67	−.07
Eat evening meal with child^b^ (0–6)	5.00		5.00		56	.28	.15	5.00		29	.79	.05
Shopping practices (0–4)	2.40	.92	2.50	.68	55	.72	.05	2.57	.82	25	.08	.37
Child fruit and vegetable consumption (0–8)	4.45	2.08	4.35	1.74	54	.80	.04	4.97	1.96	26	.68	.08
Healthy food availability (0–100)	66.82	15.62	70.12	13.98	55	.36	.12	67.12	16.30	29	.23	.23
Unhealthy food availability (0–100)	41.42	16.11	39.66	17.80	55	.02^*^	.31	33.76	19.92	28	.01^*^	.53
Child’s physical activity behaviour^b^ (0–7)	5.00		4.00		55	.44	−.10	6.00		26	.35	.18
Child’s sedentary behaviour^b^ (0–6)	1.00		1.00		48	.08	−.25	1.00		23	.58	.12
Caregiver physical activity behaviour^b^ (minutes)	100.00		110.00		48	.31	.15	82.00		26	.15	−.28
Social Support												
Physical activity	3.10	.92	2.91	.83	58	.16	.19	3.29	.88	58	.12	.31
Healthy eating	4.08	1.00	4.28	.87	64	.32	.13	4.30	.87	31	.20	.23
Healthy Related Quality of Life	71.64	16.52	74.66	9.42	20	.56	.14	75.62	16.75	14	.57	.24

^a^Values reported in the M column represent percent correct based on all that answered the question. *p* values calculated using Exact McNemar’s chi-squared test, *effect size* calculated as the odds ratio. Odds ratios were calculated based on the chances of obtaining the correct answer following Healthy Together if your answer was incorrect before the program

^b^Values reported in the M column represent median score. *p* values calculated using Wilcoxon sign-test, *effect size* calculated as *r*

^*^Significant difference between time points, *p < .05*

##### Efficacy beliefs

Immediately following HT, caregivers were more confident that they could engage in healthy eating practices, *t*(64) = −2.37, *p* = .02, *d* = .32. This increase was maintained 6-month follow-up, *t*(31) = −2.25, *p* = .03, *d* = .47. Individual site analyses revealed that caregivers from site B showed increased confidence to engage in healthy eating practices immediately after HT, *t*(5) = 3.16, *p* = 0.25, *d* = .27. This increase was not maintained at 6-month follow-up, though only 2 individuals completed both time-points. There was a significant increase in caregiver’s confidence to cook immediately following HT, *t*(63) = −3.40, *p* = .001, *d* = .43, this increase was maintained at 6-month follow-up, *t*(31) = −2.78, *p* = .01, *d* = .49. Individual analyses showed that caregivers at site C showed increased confidence to cook both immediately after HT and at 6-month follow-up (*ps* < ..05, *ds >* .57). Immediately after HT there was a significant increase in caregiver’s confidence to involve their child in cooking, *t*(61) = −2.02, *p* = .05, *d* = .32. No change in confidence to engage children in cooking was found between pre-HT and 6-month follow-up or amongst sites individually (*p* = .50). There were no changes in confidence to engage in physical activity from pre- to post-program or from pre to 6-month follow-up (*ps* > .05). However, individual site analyses revealed a significant increase in confidence to engage in physical activity for sites B and J from pre-post HT, *t*(5) = −2.93, *p* = .03, *d* = .44 and *t*(11) = −3.541, *p* = .01, *d* = 1.04 respectively. Site J appeared to marginally maintain this increase at 6-month follow-up, *t*(11) = −2.115, *p* = .06, *d* = .55.

##### Behaviour

Caregivers self-reported shopping practices did not change post HT or at 6-month follow-up compared to pre HT, nor did the availability of healthy food in the house (*ps* > .05). There was, a significant decrease in the availability of unhealthy food immediately after the program (*t*(54) = 2.37, *p* = .02, *d* = .31) and at 6-month follow-up (*t*(27) = 2.79, *p* = .01, *d* = .53) compared to pre-program. Analyses of individual sites revealed a significant increase in healthy shopping practices pre HTand at 6-month follow-up for sites D and I (*ps* < .05, *ds >* .49). Site A showed a decrease in the availability of unhealthy food immediately post program, *t*(7) = 2.78, *p* = .03, *d* = .67. Maintenance statistics were unavailable due to the limited sample size. While Site J showed a decrease in the availability of unhealthy food from before the program to 6-month follow-up, *t*(8) = 2.89, *p* = .02, *d* = .57. Caregivers reported no changes in their children’s fruit and vegetable intake, their personal fruit and vegetable intake or their personal breakfast consumption after HT (*ps* > .05). No changes in the frequency of consuming an evening meal with their child were reported following HT (*ps* > .05). No changes were found in caregiver’s reports of children’s physical activity behaviour or screen time hours outside of school hours or in caregiver’s personal physical activity post HT or at 6-month follow-up compared to pre-intervention (*ps* > .05). Individual analyses revealed that caregivers in site A reported a decrease in children’s physical activity from before (*Mdn* = 7) compared to after the program (*Mdn =* 4), Z = −2.06, *p* = .04, *r* = .73.

##### Social support

There were no changes in parental social support provided for physical activity or healthy eating immediately after HT or at 6-month follow-up compared to baseline (*ps* > .05). However, site A showed a significant decrease in social support for physical activity immediately following the program, *t*(6) = 2.68, *p* = .04, *d* = .78. Insufficient sample size eluded examination of social support for physical activity at 6-month follow-up for site A.

##### Health related quality of life

No changes were reported in parental reports of children’s’s total health related quality of life after HT or at 6-month follow-up compared to pre-intervention (*ps* > .05). No differences in health related quality of life were found within sites.

#### Children outcomes

##### Knowledge

There were no short (post-program) or long-term (6-month) changes in children’s knowledge of the daily fruit and vegetable requirements or sugary drinks (*ps* > .05). Knowledge of the recommended number of minutes of daily physical activity increased from pre- to post-program, χ^2^(1) *=* 4.65*, p* = .03, OR = .21. This increase was not seen between pre-program and 6-month follow-up (*p* = .18). Knowledge of screen time recommendations was not changed (*ps* > .05; see Table 3 for pre, post and 6-month follow-up changes for each outcome variable). No differences in knowledge were found within individual sites (*ps* > .05) (Table 3).

**Table 3:** Changes in outcome variables for children and youth

Measure	Pre-Intervention		Post-Intervention					6-month follow-up				
	*M*	SD	*M*	SD	*n*	*p*	*es*	*M*	SD	*n*	*p*	*es*
Knowledge^a^												
Daily fruit and vegetable requirement	15.1 (22)		22.8 (18)		75	.65	.25	23.8 (10)		42	.55	.33
Sugary drinks (Module 3 only)	85.2 (69)		92.9 (39)		41	.45	.30	90.3 (28)		27	1.0	−.13
Daily physical activity	43.8 (64)		55.8 (43)		76	.03*	.21	62.2 (28)		43	.18	.20
Screen time limitations	24.5 (34)		35.5 (27)		72	.52	.27	38.6 (17)		39	.09	.29
Efficacy												
Moderate-to-vigorous physical activity (0–100)	67.10	26.74	68.96	22.39	68	.08	−.21	67.90	25.29	33	.77	−.05
Fruit and vegetable consumption^b^ (1–5)	3.50		3.50		65	.01*	.33	4.0		29	.52	.12
Eat breakfast^b^ (1–5)	4.00			4.50	66	.39	.11	4.50		30	.34	−.17
Choose healthy drinks^b^ (1–5)	4.00		5.00		66	.56	.07	4.00		30	.82	−.04
Cooking efficacy (1–5)	4.00		4.33		62	.45	.10	4.17		30	.78	.05
Behaviour												
Consumption of fruits and vegetables (1–7)	4.06	1.64	3.88	1.63	44	.54	.09	4.08	1.77	27	.78	.05
Consumption unhealthy food (1–7)	2.54	2.41	2.41	1.22	44	.98	.004	2.51	.89	26	.72	.07
Consumption of regular pop^b^ (1–7)	2.00		2.00		52	.45	−.10	2.00		30	.08	−.32
Eat breakfast^b^ (1–5)	4.00		4.00		51	.93	.01	4.00		29	.10	.31
Help cook^b^ (1–5)	3.00		3.00		50	.44	.11	3.00		28	.28	.21
Hours per day in front of screen (0–6)	5.43	3.69	4.96	3.45	54	.58	.08	5.45	2.76	29	.67	.08
Num of days spent in MVPA 30 + mins	2.00		3.00		53	.78	.04	3.00		28	.89	.03
Social Support (0–4)												
Physical activity	2.45	1.10	2.63	1.22	59	1.00	0	2.60	1.26	28	.88	.03
Healthy eating	3.38	1.22	3.56	1.34	57	.29	.14	3.37	1.22	29	.49	.13
Health Related Quality of Life (0–100)	71.12	15.82	73.16	16.82	56	.08	.21	73.42	15.31	29	.36	.15

^a^ Values reported in the M column represent percent correct based on all that answered the question. The number of responses in provided in parenthesis. *p* values calculated using Exact McNemar’s chi-squared test, effect size calculated as the odds ratio. Odds ratios were calculated based on the chances of obtaining the correct answer following Healthy Together if your answer was incorrect before the program

^b^Values reported in the M column represent median score. *p* values calculated using Wilcoxon sign-test, *effect size* calculated as *r*

^*^Significant difference between time points, *p < .05*

##### Efficacy beliefs

Immediately after HT and at 6-month follow-up children reported no changes in their confidence to engage in physical activity compared to baseline (*ps* > .05). Individual site analyses revealed that children at site B were more confident to engage in physical activity at 6-month after HT compared to before, *t*(2) = −5.05, *p* = .04, *d* = 2.89. Children were 0.33 times more confident that they could consume fruits and vegetables immediately after HT (*Mdn* = 4.0) compared to before HT (*Mdn* = 4.0), *z* = 2.70, *p* = .01. Individual analyses revealed that children specifically from site C were 0.85 times more confident that they could consume fruits and vegetables immediately after HT (*Mdn* = 2.75) compared to before HT (*Mdn* = 3.5), z = −2.55, *p* = .01. No changes were reported in confidence to eat breakfast, choose healthy drinks or cook from pre- to post-HT or pre-HT to 6-month follow-up for the group as a whole or by site.

##### Behaviour

No changes were reported in children’s self-reported fruit and vegetable consumption, consumption of unhealthy foods or pop intake, frequency of breakfast consumption or frequency of assisting with cooking immediately after HT or at 6-month follow-up compared to baseline for the whole group (*ps* > .05). Individually, children from site A showed an increase in the consumption of unhealthy food from before to after HT, *t*(8) = −2.32, *p* = .49, though the effect size was small (*d* = .12). Children at site I showed a decrease in their consumption of unhealthy food from pre to post program, *t*(3) = 4.38, *p* = .02, *d =* 3.31. Children in Site H showed a reduction in unhealthy food consumption from before to 6-months after the program *t*(7) = 2.755, *p* = .03, *d =* 1.11 and a decrease in the consumption of pop intake from pre (*Mdn* = 3.5) to immediately after HT (*Mdn* = 3; z = −2.07, *p* = .04, *r* = −.69). Children did not show any changes in screen time or physical activity behaviour following HT as a whole or when assessed as individual sites (*ps* > .05).

##### Social support

No changes in perceived social support for physical activity or healthy eating were reported from pre- to post-program or from pre-program to 6-month follow-up (*ps* > .05). Children from site A reported an increase in perceived social support for both physical activity and healthy eating immediately after the program, *t*(12) = −2.28, *p* = .041, *d* = .35 and *t*(12) = −2.28, *p* = .04, *d* = .30 respectively. The sample size did not permit analyses of site A at 6-month follow-up.

##### Health related quality of life

Total health related quality of life did not change between pre- and post-program or pre-program and 6-month follow-up for the group or as individual sites (*ps* > .05).

#### Director and facilitator feedback

Facilitators believed that HT was well received by participants. One facilitator commented after session 3: “I really feel this program is making a difference to the people involved. Two families walked last week instead of driving. They are trying foods they would have never trying and enjoyed them. Therefore, I see this program as a success already”. Another commented after the final session, “Families talked about changes they have made since starting the HT program. One mom said she even started making a shopping list and she said she never does lists for anything”.

Facilitators believed that HT helped strengthen familial relationships and develop new relationships with individuals in the program. For example, one facilitator commented: “Oh, I thought it was great. Yes, definitely, having the mom and the dad and the kid come in and participate and have fun. With the teenagers it was like the moms and the daughters and it was so nice to see them like sit down, cook together. One of the girls she said like 'My mom always cooks I never cook' so her mom was like 'go, go, try it' so she cooked her first meal there. It was an amazing program”. While another facilitator stated; “I think it was a huge impact not only for the physical benefits but also the social benefits. These are newcomers [to the country] and they got to meet with other newcomers. They got to visit grocery stores. They got to do different activities that I think were purely beneficial”. Involving both the caregiver and child in the program was perceived as the biggest benefit of the program with one facilitator stating; “Families are learning to work together and be able to know and understand that if you want to be healthy you got to do different things, it's a lifestyle change”.

Directors felt that the program had a positive impact on participants while they were attending but were unsure of the long-term impact of HT due to the length of the program; “I think this is a great program with a lot of potential. However, I feel that long-term change will require a longer time period. If this program was part of a curriculum that took place over the school year, then I feel that there would be a better chance for long term change”. This feeling was echoed by the facilitators who felt that the program might be more effective if it was longer, with 10 sessions being repeatedly mentioned as a potentially viable number.

### Adoption

#### Setting level

One hundred community sites were approached to participate in the implementation of HT for which there were 10 funded positions available over 2 years. Of the 100 sites that were approached to participate 35 reported being interested in implementing HT. It is unclear how these 35 sites distinctly differed from the 65 sites that were not interested in participating in the implementation of HT.

Of the 35 sites that expressed interest in HT several sites were unable to participate due to either limited administration and personnel capacity at the time of commitment or they felt that the group learning model was not feasible for implementation at their site (i.e., hospital, day care centre). Of those sites that could commit to implementing the program, 10 were selected by The Bridge to include a representation of diverse community settings for program implementation. This included a) children in care, b) children with special needs, c) families of Aboriginal, Inuit, and/or Metis descent, d) immigrants and refugees, e) families living in rural/remote, areas, f) families living in northern communities, and g) families with low income.

#### Staff level

Data on staff recruitment was available from 9 of the 10 sites (see Additional file 7 for staff adoption data by site). Seven of the nine sites utilized staff already working for the organization to implement HT. This was possible as the employees were part-time and wanted additional hours. In addition, implementing HT was not a full-time position and coordinators felt it would have been a challenge to attract staff from outside the organization. Of these 7 sites, two approached specific staff members to be part of HT while the other five encouraged interested staff members to apply. The remaining two sites hired individuals from outside of the organization to implement HT, as the workload of their current staff was too high to ask them to implement HT in addition to their other roles.

Four of the nine sites had staff members within their organization who were not interested in implementing HT. These individuals were not interested in being part of HT as they were already employed full-time as counsellors or the program did not fit their qualifications (i.e., they did not work with children) or area of interest.

Three of the 10 site directors felt that their facilitators were typical of the staff working at their site. One site felt that their facilitators were those that enjoyed group-based work, which was not common within their child welfare agency. Healthy Together facilitators had more experience working with children and facilitating groups than the other organization staff members and had more interest in general health. One director commented; “The staff who are involved are more curious and positive and adventurous -  all interested participants have a stake in being healthy”*.* The majority of facilitators reported that they had been involved in implementing nutrition and/or healthy eating programs in the past, while only two facilitators reported having implemented any physical activity related programs in the past. Staff demographics are shown in Additional file 8 as a group and in Additional file 9 facilitators demographics are provided by site. Sites D, H and J had at least one facilitator with a post-graduate degree, while site D facilitators identified as being from a range of ethnic backgrounds including south and southeast Asian, Filipino, Black and Latin American. There appears to be a link between the demographic of staff or their experience and individual site effectiveness, although data is limited. In total 19 volunteers assisted with the HT program, contributing a total of 269.5 h of their time.

### Implementation

#### Degree to which program was implemented as per program manual

On average, program objectives were met 72.8% of the time across all modules (see Additional files 10, 11 and 12 for specific module and session implementation outcomes). The cooking activity was conducted 99.3% of the time while, other activities, including physical activity, were conducted less frequently (average 73.6%). Seven facilitators felt that they implemented HT as outlined by the program manual, while facilitators at two sites felt that they were unable to implement the program as laid out by the manual. These two sites felt that the material was not appropriate for their population due to either financial circumstances or past trauma, therefore the content was heavily reduced. Due to bad weather one site had to cancel two sessions for Module 3 and one session for Module 1. These sessions were not made up at a different time.

#### Program adaptations

Approximately 71 adaptations were made to HT across all three modules and all sessions. Twenty four adaptations involved providing alternative handouts to those included in the manual and another 24 related to using different recipes to suit the group (i.e., stove top bannock). Eight adaptations related to changing the order of the session activities. Three adaptations involved omitted activities, specifically site E did not conduct the physical activity during one session and site B did not cover the topic of family traditions due to fears of emotional distress to participants. Ten adaptions involved added components to HT, for example demonstrations of the sugar in drinks using sugar cubes by site C and bringing in a yoga instructor to teach the children basic yoga poses at site E. Two adaptations were made that were unspecified.

#### Barriers to implementation

All facilitators highlighted time constraints as the hardest element to overcome when implementing HT. Facilitators felt that the content of the program was too extensive for the 2-hour time period in which implementation was meant to occur. This restricted the facilitators from delivering HT in its entirety or developing appropriate adaptations. One facilitator commented; “Time. It was really hard to fit in everything that you were expected to fit in, in each module in the time that was allotted”*.* Due to the extenstive content particiapants were reportedly overwhelmed with the amount of information; “I think sometimes though by the end of the um, material they were a little overloaded with just the amount of content that they— the information they were given in one session”*.* Faciliators felt that the literacy level of the content and handouts, compared to the literacy levels of their participants was a barrier to providing some of the recommended handouts; “I think for me um, just ethically when I'm presenting information I'm always conscious of literacy levels. So at some point I did struggle with how we were presenting information, and just knowing some of the barriers that our families faced, following through on homework and stuff like that? I was a little hesitant with some of the material”. In addition, some cultural and social factors arose within the groups that affected how the facilitator implemented HT. These included; religious-based food restrictions impacting usability of recipes, the family traditions program content for children in care and concerns regarding the use of the word ‘family’ and the economic struggles faced by some of the families and facilitators feeling uncomfortable discussing purchasing certain types of food. A number of quotes highlight these concerns; "We have some families that, like, for like, religious reasons they don't eat certain foods", and "We were very cognitive that we had children in care, and some of their triggers. We cut out parts of the program based on that". In addition some facilitators commented, "For me I struggled with the economics of, and the financial situations of many of our families. They might want to eat healthier but might not be able to afford the healthy fresh vegetables, and fruits, and that kind of thing. So not that they don't want to, but just can’t with what they’re given".

#### Facilitators to implementation

Facilitators felt the HT manual was easy to follow and contained a lot of good information; "Very well organized and prepared, easy to just take the book and be able to do the program with the family and the kids". In addition, many facilitators felt that they could adapt the program to their communities needs such as including local fruits and vegetables to their region or adapting recipes to accommodate religious needs. Facilitators with more experience reported a greater ease in modifying the content then those with less experience.

#### Costs – Time

The original manual suggested that facilitators allocated 6 h per week to prepare for and deliver HT, while program assistants should allocate 4 h. On average facilitators spent 6.41 h per week prepping and delivering HT, while program assistants spent 4.28 h per week on average prepping for HT. Facilitators reported that preparation took longer for HT as they had never delivered the program before and because they sought out alternative resources to those provided within the manual.

#### Costs - Financial

As part of the project each site was allocated $36,000 to implement the program. This money was provided to cover staff wages and program supplies, including physical activity materials and cooking ingredients. Only two sites provided information regarding the cost of running HT. One site reported that the program cost the full $36,000 to implement with the majority of money spent on staff wages ($27,412). Additional funds were spent on purchasing resources ($3302) and miscellaneous costs including insurance, computers, promotion, rent and transportation ($5958). The second site reported the program costing $14,059 to implement. Costs included staff wages ($12,362) and resources ($2141). Directors noted that HT cost a significant amount of money to implement given the cost of cooking ingredients and physical activity resources. In addition, some sites had to rent spaces in which to conduct the cooking component of the program. The majority of sites felt that this program was not sustainable without additional funding.

### Maintenance

#### Setting level

Two implementation sites completed HT in full in the year following initial program implementation (see Additional file 13 for maintenance data based on site). Five sites incorporated components of HT into existing programs. One site had not implemented HT in the previous year, or aspects of it, due to time constraints and leadership changes but planned to do so in the upcoming year with residual funds from the original implementation. Many directors and program coordinators felt that HT would not be feasible to run in its entirety without funding, with the majority of funds being required for staff wages. In general directors felt that HT could be incorporated into existing programs to reduce costs. One director felt that HT did not align with the needs of their target population, specifically a trauma-exposed population, while all others felt that HT aligned with their organizational mission.

## Discussion

The current study utilized the RE-AIM framework to evaluate the internal and external validity of a community-developed and implemented healthy eating and physical activity program designed for vulnerable children and their families. The evaluation identified program strengths as well as important areas for improvement and demonstrates the challenges of conducting community-based research.

### Reach

Healthy Together (HT) reached a small portion of eligible families. Although all sites stated serving vulnerable populations as part of their organization mission only two sites expressed definitive eligibility criteria beyond the HT age requirements. This was due to low response rates from the target populations, leading to the expansion of recruitment to all families within the sites catchment areas. Broadening the target population limits inferences regarding the reach and impact of HT on the vulnerable populations for which it was developed. While recruiting children for community programs is challenging [26] it is recommended that program developers and implementers clearly define the target population before recruitment in order to establish if the program is able to reach and positively impact the specified population. In addition to recruitment challenges, high attrition rates were reported, similar to those seen in previous research with children of ethnic minorities and from low socio-economic families [27]. The challenge of recruiting and retaining children from vulnerable populations for community programs are not uncommon but are rarely reported by primary intervention studies [28, 29]. It is imperative that community sites make a concerted effort to employ directed strategies to recruit, engage and retain children and their families for such programs such as establishing trusting relationships with parents and children, utilizing a program champion and offering participation incentives [30].

### Effectiveness

HT increased caregiver’s confidence to engage in healthy eating practices and to cook. Furthermore, caregivers reported a reduction in the availability of unhealthy food available in the house immediately after HT and at 6-month follow-up, replicating the finding of Robertson and colleagues [31] in their 12-week family focused community program. When examined by site, Sites A and J appeared to demonstrate reductions in unhealthy food availability between pre- to post-program (Site A) and pre-program to 6-month follow-up.

HT did not impact self or proxy reports of individual behaviour, perceived social support for physical activity and healthy nutrition or the broader outcome of quality of life. One explanation for the lack of changes to behaviour, social support, and quality of life in general is the atheoretical nature of the program. A recent literature review demonstrated that theory-based interventions are more successful than atheoretical approaches in changing adolescent’s physical activity behaviour [32]. Similarly, Hoelscher and colleagues [33] highlight the importance of theory in designing effective nutrition interventions for adolescents. Interventions created without a guiding theoretical framework elude examination and understanding of the causal mechanisms involved in complex behaviour change such as nutrition and physical activity [34]. Furthermore, without explicitly targeting theoretical constructs of change establishing the most appropriate channels of program adaptation are challenging [32]. An additional factor that may limit the effectiveness of HT is the short-term nature of the program. Specifically, five 2-hour sessions may not have been sufficient to evoke long-term changes in the desired cognitive or behavioural outcomes. Similar community-based family obesity prevention programs conducted over a greater time period have reported positive outcomes [15, 35]. It is recommended that future community-based behaviour change programs work closely with behaviour change experts to co-develop theoretically driven content and establish appropriate program duration to maximize the likelihood of promoting change in outcomes of interest. While the overall impact of any health program cannot be predicted by effectiveness alone [36], it should be taken into consideration when contemplating the future of a program.

### Implementation

In this iteration of HT (version 1), fidelity was moderate with facilitators stating *time* as the biggest barrier to program implementation. Due to limited time, and the large amount of content contained within HT, facilitators felt unable to implement all components. Observations from site A revealed that the percentage to which session objectives were met was low in a number of sessions due to the facilitators dropping aspects of the program due to time, often the physical activity section. Interestingly, in site A caregivers reported a decrease in children’s physical activity behaviour from before to after HT as well as a decrease in the amount of social support they provided. The degree to which nutrition-related components, such as cooking, were completed was 20% higher than physical activity related components. Given that implementation fidelity can influence program effectiveness [37], higher fidelity could explain the more favorable outcomes reported for nutrition related outcomes in caregivers in comparison to physical activity outcomes. Differences in implementation fidelity between nutrition and physical activity content could be due to the lack of experience of the facilitators with the program content. Specifically, the majority of facilitators reported previous involvement in nutrition programs; however, only 2 facilitators had previously facilitated physical activity programs. It is possible that, given the time constraints, facilitators chose to drop the physical activity portion of the program ahead of the cooking section due to comfort in facilitating the activity. The knowledge, experience and confidence of facilitators in delivering a program and its content are essential to the success of a program [38, 39]. It is imperative that the experience and confidence level of potential staff be examined in order to plan appropriate training. Prior to facilitating HT facilitators received 2 days of training that focused heavily on the process of group facilitation. Community-based programs targeting nutrition and physical activity are encouraged to work with nutrition and physical activity experts to provide facilitators with training on program content to ensure facilitators feel knowledgeable and confident to implement the program as intended [15, 31, 35]. Given the extensive content within HT, facilitators and directors believed that program effectiveness and fidelity might benefit from extending the length of the HT program, which the Bridge now raise as an option in their current HT facilitator trainings. Similar family community-based obesity prevention programs have ranged from 9-weeks [15] to a 4-week camp [35], with positive outcomes.

### Maintenance

Due to the perceived high cost of implementing HT many sites incorporated specific components of the program within other existing programs in the follow-up year. Community sites are tightly bound by financial constraints and determining methods to reduce costs is essential if a program is to be sustained. Sites that worked in close collaboration with community partners, such as schools, were able to reduce costs by utilizing school facilities. Furthermore, collaboration with schools assisted in the recruitment of a number of participants. The development of community partnerships has been highlighted as essential in ensuring the success of a program [40] and as such HT developers are providing future implementation sites with suggestions on how to engage potential community partners.

### Strengths and limitations of the study and community based program evaluation

As is common when conducting community-based interventions [40, 41], lack of reliable data and a control group was a constraint in this evaluation and highlights the need for caution when reviewing findings. However, the use of multiple data sources to assess each RE-AIM component in this study provided a substantial amount of rich data, which serves to increase confidence in the conclusions made, and adds contextual relevance to the findings. A second limitation is the inability to assess intervention dose response due to limited monitoring of participant attendance at program sessions and the ‘drop-in’ format employed by some sites. A third limitation was the high rate of attrition, restricting full examination of outcome variables by site, as well as multi-level modeling to account for the nested populations. While effectiveness was examined by site where possible the inability to fully examine effectiveness of HT at every site makes it extremely difficult to identify links between site specific contextual factors and program effectiveness. Data pertaining to the individual sites should be considered with caution given the limited sample size and the potential for Type 1 error. The lack of a control arm meant no causal inferences could be made. Ideally, more robust research, such as a randomized controlled trial, is needed in order to determine the effectiveness of HT under ideal conditions. That being said, evaluation of real world community programs enables the examination of external factors regularly neglected in rigorous intervention studies [42]. The current evaluation examines both external and internal indicators of program success in an attempt to provide a broad understanding of the impact of HT. It is clear that a balance needs to be established between the interests of the community and the desire for rigorous evaluation methodology [43].

### The future of healthy together

This is the first evaluation of the first iteration of HT, a family-education program designed and implemented by a community organization to address healthy weights in vulnerable children. Given the growing evidence in support of caregiver-inclusive interventions to address pediatric obesity [44] HT has the potential to make a positive impact on its target audience if modications are made. This evaluation provides the opportunity for HT developers, and other community-based organizations, to address areas of concern in order to strengthen the overall impact of the program before further dissemination and implementation. Based on these findings the Bridge have made a number of adaptations to HT, including a) providing facilitators with training on the specific program content in order to increase knowledge and confidence of program delivery and b) increasing the length of the program from 5- to 10-weeks in order for all program content to be covered.

## Conclusion

Overall Healthy Together represents a feasible community-based healthy weights initiative that can be successfully implemented in a variety of populations across Canada. However, further refinement of the program is required in order to ensure the program is effective at positively impacting physical activity and healthy eating cognitions and behaviour. In is recommended that community organizations, such as the Bridge, work closely with behaviour change experts to ensure that community designed interventions target key theoretical constructs found to promote behaviour change. In addition, program implementation and dissemination experts should be consulted to increase potential of the program to positively impact public health. Simultaneously the continued evaluation from arms-length evaluators that examine changes in outcomes of interest through both qualitative and quantitative means is warranted.

## Additional files

Additional file 1: *Overview of measures used in the Healthy Together evaluation, data collected and timeline.* Description of what measures were taken and when during the Health Together evaluation. (DOCX 59 kb)

Additional file 2: *Description of Child Survey.* Explanation of the items used within the child and youth surveys and associated reliability statistics. (DOCX 18 kb)

Additional file 3: *Description of Caregiver Survey*. Explanation of the items used within the caregivers surveys and associated reliability statistics. (DOCX 19 kb)

Additional file 4: *Individual site analyses and details of which sites had sufficient data to perform analyses (caregiver and child)*. (DOCX 25 kb)

Additional file 5: *Statistics related to the Reach of the Healthy Together program.* Description of data used examine program reach. (DOCX 14 kb)

Additional file 6: *Demographic characteristics of the caregivers at baseline based on site*. (DOCX 21 kb)

Additional file 7: *Staff adoption of Healthy Together.* Description of internal and external recruitment for Healthy Together across sites. (DOCX 54 kb)

Additional file 8: *Healthy Together staff demographics*. (DOCX 47 kb)

Additional file 9: *Facilitator demographics by site*. (DOCX 15 kb)

Additional file 10: *Evaluator’s Observations Module 1 (Data from 8 implementation sites)*. Description of specific module and session outcomes for Module 1 based on observations. (DOCX 16 kb)

Additional file 11: *Evaluator’s Observations Module 2 (Data from 10 implementation sites).* Description of specific module and session outcomes for Module 2 based on observations. (DOCX 16 kb)

Additional file 12: *Evaluator’s Observations Module 3 (Data from 10 implementation sites).* Description of specific module and session outcomes for Module 3 based on observations. (DOCX 16 kb)

Additional file 13: Setting level maintenance of Healthy Together in the past year. (DOCX 15 kb)
