# Supplementary material for: Evaluation of a community-based, family focused healthy weights initiative using the RE-AIM framework
Source: Int J Behav Nutr Phys Act. 2018 Jan 26;15:13. doi: 10.1186/s12966-017-0638-0 (PMC5787319; doi:10.1186/s12966-017-0638-0)
Supplement: Supplementary file 1 — Overview of measures used in the Healthy Together evaluation, data collected and timeline. Description of what measures were taken and when during the Health Together evaluation. (DOCX 59 kb) [file 12966_2017_638_MOESM1_ESM.docx]

| **Additional File 1.** Overview of measures used in the Healthy Together evaluation, data collected and timeline | | | | | | | | | |
| --- | --- | --- | --- | --- | --- | --- | --- | --- | --- |
|  | **Pre Program** | **Session 1** | **Session 2** | **Session 3** | **Session 4** | **Session 5** | **1-week post program** | **6-month** | **1-year** |
| **Children and Youth**  **7-18** | Questionnaire (E) |  |  |  |  |  | Questionnaire (E) | Questionnaire (E) |  |
| **Caregivers** | Questionnaire (E) |  |  |  |  |  | Questionnaire (E) | Questionnaire (E) |  |
| **Facilitators** | Survey (A) | Observation (I)  Post session survey (I) | Observation (I)  Post session survey (I) | Observation (I)  Post session survey (I) | Observation (I)  Post session survey (I) | Observation (I)  Post session survey (I) | Group Interviews (E, I) |  |  |
| **Coordinator** | Survey  (R, A) |  |  |  |  |  | Survey (R)  Summary Forms (R) |  | Survey (M)  Emails (M) |
| **Director** | Survey (A)  One-on-one Interviews (R, A) |  |  |  |  |  | One-on-one Interviews (E) |  | Survey (M)  Emails (M) |
| **The Bridge** |  |  |  |  |  |  | Documentation (A, I) |  |  |
| **Statistics Canada** |  |  |  |  |  |  | Archival records (R) |  |  |
| Note. The letters appearing after each measure in parenthesis correspond to the RE-AIM construct that the measure is assessing. *R* = reach; *E* = effectiveness; *A* = adoption, *I* = implementation; *M* = maintenance. | | | | | | | | | |
