# Supplementary material for: Evaluation of a community-based, family focused healthy weights initiative using the RE-AIM framework
Source: Int J Behav Nutr Phys Act. 2018 Jan 26;15:13. doi: 10.1186/s12966-017-0638-0 (PMC5787319; doi:10.1186/s12966-017-0638-0)
Supplement: Supplementary file 2 — Description of Child Survey. Explanation of the items used within the child and youth surveys and associated reliability statistics. (DOCX 18 kb) [file 12966_2017_638_MOESM2_ESM.docx]

| **Additional File 2.** Description of child survey | | | | | | | |
| --- | --- | --- | --- | --- | --- | --- | --- |
| **Outcome** | | **Measure** | **Items** | **Exemplar question** | **Response options** | **Scoring** | **α** |
| **Knowledge** | Daily recommended intake of fruit and vegetables | Researcher developed. Based on Canada’s food guide | 1 | How many servings of fruits and vegetables should a child your age consume each day? | 1 (At least 2) to 4 (At least 5); 9 (I don’t know) | Stand alone  Based on age response scored a correct or incorrect | N/A |
|  | Sugary drinks | Researcher developed. Based on program content | 1 | Which of these drinks contains the MOST sugar? | 1% Milk, Pop, Fruit drink, Chocolate milk | Stand alone  Response scored as correct or incorrect | N/A |
|  | Daily physical activity guidelines | Researcher developed. Based on Canada’s physical activity guidelines | 1 | How many minutes of physical activity do you think children your age should get each day to be healthy? | 1 (At least 15 minutes each day) to 4 (At least 90 minutes each day); 9 (I don’t know) | Stand alone  Responses scored as correct or incorrect | N/A |
|  | Daily screen time guidelines | Researcher developed. Based on Canada’s screen time guidelines | 1 | What is the maximum number of screen time hours (e.g., T.V., Computer, Phone) that children your age are supposed to watch per day? | 0 (Less than 1 hour) to 4 (4 hours) | Stand alone  Responses scored as correct or incorrect | N/A |
| **Efficacy** | Moderate to vigorous physical activity | Physical Activity Task Self-Efficacy (McAuley and Mihalko, 1998)  Modified version | 6 | How confident are you that you can complete 10 minutes of physical activity at a moderate intensity three times next week? | 0% (Not at all confident) to 100% (Completely confident) | Mean composite score | α’s ≥ .93 |
|  | Consume fruit and vegetables | Researcher developed. Based on program content | 2 | I think I can eat 4 servings of fruits and vegetables a day | 1 (I definitely can’t) to  5 (I definitely can) | Mean composite score | α’s ≥ .88 |
|  | Eat breakfast | Researcher developed. Based on program content | 1 | I think I can eat breakfast everyday | 1 (I definitely can’t) to  5 (I definitely can) | Stand alone item | N/A |
|  | Choose healthy drinks | Researcher developed. Based on program content | 1 | I think I can choose healthy drinks (e.g., water and milk) instead of unhealthy drinks (e.g., pop) | 1 (I definitely can’t) to  5 (I definitely can) | Stand alone item | N/A |
|  | Help cook | Researcher developed. Based on program content | 6 | I think I can cut fruits and vegetables | 1 (I definitely can’t) to  5 (I definitely can) | Mean composite score | α’s ≥ .74 |
| **Behaviour** | Consumption of fruits and vegetables | Beverage and Snack Questionnaire. Neuhouser et al., 2009  Modified version | 2 | How often did you eat these vegetables in the past week? | 1 (Never or less than 1 per week) to 7 (4+ per week) | Mean composite score | α’s ≥ .73 |
|  | Consumption of unhealthy foods | Beverage and Snack Questionnaire (Neuhouser et al., 2009)  Modified version | 5 | How often did you eat candy in the past week? | 1 (Never or less than 1 per week) to 7 (4+ per week) | Mean composite score | α’s ≥ .70 |
|  | Pop consumption | Beverage and Snack Questionnaire (Neuhouser et al., 2009)  Modified version | 1 | How often did you drink regular pop in the past week? | 1 (Never or less than 1 per week) to 7 (4+ per week) | Stand alone item | N/A |
|  | Breakfast consumption | Researcher developed. Based on program content | 1 | In the past week how often did you eat breakfast? | 1 (never) to 5 (almost always) | Stand alone item | N/A |
|  | Helped cook meals | Researcher developed. Based on program content | 1 | In the past week how often did you help cook meals? | 1 (never) to 5 (almost always) | Stand alone item | N/A |
|  | Physical activity | 4^th^ grade School Physical Activity and Nutrition Questionnaire (SPAN; Penkilo, George, & Hoelscher, 2008) | 1 | On how many of the past 7 days did you exercise or take part in physical activity that made your heart beat fast and made you breathe hard for at least 30 minutes? | 0 (0 days) to 7 (7 days) | Stand alone item | N/A |
|  | Screen time | 4^th^ grade School Physical Activity and Nutrition Questionnaire (Penkilo, George & Hoelscher, 2008) | 3 | How many hours per day do you usually watch TV, DVDs, or movies away from school? | 0 (I don’t watch TV, DVDs, or movies) to 6 (6 hours or more) | Items summed | N/A |
| **Social Support** | Physical activity | 8^th^ grade School Physical Activity and Nutrition Questionnaire (Hoelscher et al., 1999) | 6 | How often does your caregiver exercise with you? | 0 (Never) to 4 (Everyday) | Mean composite score | α’s ≥ .88 |
|  | Healthy eating | 8^th^ grade School Physical Activity and Nutrition Questionnaire (Hoelscher et al., 1999) | 3 | How often does your caregiver eat lots of fruits and vegetables with you? | 0 (Never) to 4 (Everyday) | Mean composite score | α’s ≥ .71 |
| **Health Related Quality of Life** | Total HRQOL | PEdsQL (Varni, 1999) | 23 | In the past ONE month: I feel afraid or scared | 0 (Never) to 100 (almost always) | Mean composite score | α’s ≥ .71 |
