# Supplementary material for: Evaluation of a community-based, family focused healthy weights initiative using the RE-AIM framework
Source: Int J Behav Nutr Phys Act. 2018 Jan 26;15:13. doi: 10.1186/s12966-017-0638-0 (PMC5787319; doi:10.1186/s12966-017-0638-0)
Supplement: Supplementary file 3 — Description of Caregiver Survey. Explanation of the items used within the caregivers surveys and associated reliability statistics. (DOCX 19 kb) [file 12966_2017_638_MOESM3_ESM.docx]

| **Additional File 3.** Description of caregiver survey | | | | | | | |
| --- | --- | --- | --- | --- | --- | --- | --- |
| **Outcome** | | **Measure** | **Items** | **Exemplar question** | **Response options** | **Scoring** | **α** |
| **Knowledge** | Daily fruit and vegetable requirements (adult and child) | Researcher developed. Based on Canada’s food guide | 2 | How many total cups of fruits and vegetables should you (an adult) eat each day? | 1 (At least 2) to 4 (At least 5); 9 (I don’t know) | Stand alone  Responses scored a correct or incorrect | N/A |
|  | Sugary drinks | Researcher developed. Based on program content | 2 | Which of these drinks contains the MOST sugar? | 1% Milk, Pop, Fruit drink, Chocolate milk | Stand alone  Responses scored a correct or incorrect | N/A |
|  | Physical activity guidelines *duration* (adult and child) | Researcher developed. Based on Canada’s physical activity guidelines | 2 | Experts recommend that children aged 7-12^*^ should be physically active for at least how many minutes per day? | 1 (10 minutes) to 5 (90 minutes); 9 (I don’t know) | Stand alone  Based on age responses scored a correct or incorrect | N/A |
|  | Physical activity guidelines *frequency* (adult and child) | Researcher developed. Based on Canada’s physical activity guidelines | 2 | Experts recommend that adults should be physically active on how many days per week? | 0 (0 days) to 7 (7 days); 9 (I don’t know) | Stand alone  Responses scored a correct or incorrect | N/A |
|  | Screen time guidelines (child) | Researcher developed. Based on Canada’s screen time guidelines | 1 | Experts recommend that children aged 7-12^*^ participate in no more than how many hours of media-related activities, such as TV watching and video game playing, per day? | 1 (1 hour) to 6 (6 hours); 9 (I don’t know) | Stand alone  Responses scored as correct or incorrect | N/A |
| **Efficacy** | Consume a healthy diet | Researcher developed.  Based on program content | 3 | How confident are you that you can eat healthy food when craving unhealthy food? | 1 (Not at all confident) to 5 (Completely confident) | Mean composite score | α’s ≥ .76 |
|  | Cooking efficacy | Cooking Self-Efficacy Scale (Michaud, 2007)  Modified version | 4 | How confident are you that you can cook from basic ingredients? | 0% (Not at all confident) to 100% (Completely confident | Mean composite score | α’s ≥ .84 |
|  | Include child in cooking | Researcher developed.  Based on program content | 3 | How confident are you that you can include your child in meal preparation? | 1 (Not at all confident) to 5 (Completely confident) | Mean composite score | α’s ≥ .84 |
|  | Overcome physical activity barriers | Physical activity barrier efficacy (Cramp and Brawley, 2006) | 7 | How confident are you that you would still be physically active even if you were tired? | 0% (absolutely not confident) to 100% (absolutely confident) | Mean composite score | α’s ≥ .67 |
| **Behaviour** | Eat breakfast | 2010 Parents School Physical Activity and Nutrition Questionnaire (Pérez et al., 2010) | 1 | During the past 7-days, how many times did you eat breakfast? | 0 (Never) to 6 (More than 7-times) | Stand alone | N/A |
|  | Caregiver fruit and vegetable consumption | Researcher developed. Based on program content | 2 | During the past 7-days, how many times did you eat fruit (including fresh, dried, frozen and canned)? | 0 (Never) to 6 (More than 7-times) | Mean composite score | α’s ≥ .71 |
|  | Eat evening meal with child | 2010 Parents School Physical Activity and Nutrition Questionnaire (Pérez et al., 2010) | 1 | During the past 7 days, how many times did you eat an evening meal together with your child? | 0 (Never) to 6 (More than 7-times) | Stand alone | N/A |
|  | Shopping practices (0-4) | fruit and vegetable shopping practice scale (Baranowski et al, 2006) | 5 | In the past week how often did you plan meals for the upcoming week? | 0 (Never) to 4 (Almost always) | Mean composite score | α’s ≥ .68 |
|  | Child fruit and vegetable consumption | Fruit and Vegetable Screening Measure  (Prochaska & Sallis, 2004) | 2 | In a typical day, how many servings of fruit does your child eat (serving sizes are explained) | 0 (None) to 4 (4 or more) | Mean composite score | α’s ≥ .57 |
|  | Availability of food in the home | Home food environment questionnaire (Ding et al, 2012)  Modified version | 14 | During the past week how often were the following food items available in your home?  E.g., fresh fruit, salad, candy, chips | 0 (Never) to 100 (always) | Items separated into healthy and unhealthy.  Mean composite scores created for healthy and unhealthy foods | α’s ≥ .68 |
|  | Child’s physical activity | Physical Activity Screening Measure  (Prochaska et al., 2001) | 1 | For the past seven days, how many days was your child physically active for a total of at least 60 minutes per day? | 0 (0 days) to 7 (7 days) | Stand alone | N/A |
|  | Child’s screen time | (Robinson et al, 1999) | 1 | How many hours per day does your child spend on the computer, watching TV, and playing video games when away from school? | 0 (0 days) to 6 (6 + days) | Stand alone | N/A |
|  | Caregiver physical activity | Short Form International Physical Activity Questionnaire (Craig et al., 2003) | 6 | **Frequency**: During the last 7 days on how many days did you do VIGOROUS physical activities like heavy lifting, aerobics, or fast bicycling  **Duration**: On average how long did each session of vigorous exercise last? | Open ended | Frequency x duration for each intensity.  Sum moderate and vigorous to calculate MVPA | N/A |
| **Social Support** | Physical activity | Amherst Health and Activity Study (Sallis et al., 2002) | 5 | During the past week, how often have you watched your child participate in physical activity or play sports? | 1 (Never) to 5 (Daily) | Mean composite score | α’s ≥ .74 |
|  | Healthy eating | Researcher developed.  Based on program content | 3 | During the past week, how often have you encouraged your child to eat fruits and vegetables? | 1 (Never) to 5 (Daily) | Mean composite score | α’s ≥ .77 |
| **Health Related Quality of Life** | Total HRQOL | PEdsQL parent version (Varni, 1999) | 23 | In the past ONE month, how much of a problem has your child had with… walking more than one block | 0 (Never) to 100 (Almost always) | Mean composite score | α’s ≥ .84 |
| ^*^ The age range reflects the module for which the caregiver is participating, in the above instances module 2 (7-12 years). For caregivers participating in module 1 the question would reference children 0-6 years. For caregivers participating in module 3 the question would reference youth 13-18 years. | | | | | | | |
