# Supplementary material for: Evaluation of a community-based, family focused healthy weights initiative using the RE-AIM framework
Source: Int J Behav Nutr Phys Act. 2018 Jan 26;15:13. doi: 10.1186/s12966-017-0638-0 (PMC5787319; doi:10.1186/s12966-017-0638-0)
Supplement: Supplementary file 4 — Individual site analyses and details of which sites had sufficient data to perform analyses (caregiver and child). (DOCX 25 kb) [file 12966_2017_638_MOESM4_ESM.docx]

| **Additional File 4.** Individual site analyses and details of which sites had sufficient data to perform analyses (caregiver and child) | | | | |
| --- | --- | --- | --- | --- |
|  | **Individual sites with sufficient data to conduct individual level analyses** | | |  |
| **Caregivers** | **Pre-post** | **Pre-6-month** | **Findings** | |
| **Knowledge^a^** |  |  |  | |
| *Adult daily fruit and vegetable servings* | A,B,C,D,H, I, J | B,C,D,H,J | No significant differences within sites | |
| *Child daily fruit and vegetables servings* | B,C,F,H,I,J | C,H,I,J | No significant differences within sites | |
| *Sugary drinks* | A,B,C,D,F,H,I,J | B,D,H,I,J | No significant differences within sites | |
| *Adult minutes of PA per day* | A,B,C,D,F,G,H,I,J | B,C,D,H,I,J | No significant differences within sites | |
| *Child minutes of PA per day* | B,C,F,H,I,J | C,D,H,I,J | No significant differences within sites | |
| *Adult days of PA per week* | A,B,C,D,F,G,H,I,J | B,D,F,H,I,J | No significant differences within sites | |
| *Child days of PA per week* | A,B,C,D,F,G,H,I,J | B,C,D,H,I,J | No significant differences within sites | |
| *Child screen time limits* | A,B,C,D,F,G,H,I,J | B,C,D,H,I,J | No significant differences within sites | |
| **Efficacy Beliefs** |  |  |  | |
| *Adult healthy eating efficacy (1-5)* | A,B,C,D,F,G,H,I,J | B,C,D,H,I,J | **Site B:**  Significant decrease between pre - post:  t(5)=3.16, *p* = 0.25, *d* = .27  Pre mean = 2.83 (SD: .09), Post mean = 2.61 (SD: .09) | |
| *Cooking efficacy (0-100)* | A,B,C,D,F,H,I,J | B,C,D,I,J | **Site C:**  Significant increase from pre-post:  *t*(12)=-3.048, *p* = .01, *d* = .58 (Pre mean= 81.54 [17.78], post = 90.19 [12.88]) and from pre-6mfu: *t*(8)=-2.251, *p* = .05, *d* = .57 (Pre mean = 79.17 [22.53], 6mfu mean = 88.89 [11.87]). | |
| *Efficacy to include child in cooking (1-5)* | A,B,C,D,F,H,I,J | C,D,I,J | No significant difference between sites | |
| *Adult physical activity efficacy* | A,B,F,H,I,J | B,I,J | **Site B:**  Significant increase from pre to post, *t*(5) = -2.93, *p* = .03, *d* = .44, (Pre mean = 48.57 [SD: 25.05], Post mean = 57.85 [SD: 21.52])  **Site J:**  Significant increase in pre to post, *t*(11) = -3.541, *p* = .01, *d* = 1.04, (Pre mean = 44.4 [SD: 16.48], post mean = 58.45, [SD: 11.38]).  Marginally significant pre to 6m, *t*(11) = -2.115, p = .06, *d* = .55, (Pre mean = 43.81 [SD: 17], 52.26, [15.11]). | |
| **Behaviour** |  |  |  | |
| *Eat breakfast^b^ (0-6)* | A,B,C,D,H,I,J | C,D,H,I,J | No significant differences within sites | |
| *Caregiver fruit and vegetable consumption^b^ (0-6)* | A,B,C,D,H,I,J | C,D,H,I,J | No significant differences within sites | |
| *Eat evening meal with child^b^ (0-6)* | A,B,C,D,H,I,J | C,D,H,I,J | No significant differences within sites | |
| *Shopping practices (0-4)- t-tests* | A,B,C,D,F,G,H,I,J | B,C,D,F,G,H,I,J | **Site D:**  Significant increase between pre and 6m, *t*(5) = -3.67, *p* = .02, *d* = .49 (Pre mean = 2.52 [0.74], 6m = 2.87 [0.81]  **Site I:**  Significant increase between pre and 6mfu, *t*(1) = -45.44, *p* = .01, *d* = 2.02 (pre mean = 2.38 [0.59], 6m = 3.2 [0.56]. | |
| *Child fruit and vegetable consumption (0-8)- t-test* | A,B,C,D,H,I,J | C,H,I,J | No significant differences within sites | |
| *Healthy food availability (0-100)* | A,B,C,D,H,I,J | C,D,H,I,J | No significant differences within sites | |
| *Unhealthy food availability (0-100)* | A,B,C,D,H,I,J | C,D,H,I,J | **Site A:**  Sig decrease from pre-post, *t*(7)=2.78, *p* = .03, *d* = .67 (Pre mean = 42.86 [10.45], post mean = 33.48 [18.4])  **Site J:**  Significant decrease from pre-m6fu, *t*(8)=2.89, *p* = .02, d = .57 (Pre mean = 26.98 [18.39], m6fu = 17.86 [15.15] | |
| *Child’s physical activity behaviour^b^ (0-7)* | A,B,C,D,H,I,J | C,H,I,J | **Site A**  Significant decrease in PA, Z = -2.06, *p* =.04, *r* = -.73 (Pre Mdn = 7, Post Mdn = 4) | |
| *Child’s sedentary behaviour^b^ (0-6)* | A,B,C,D,H,I,J | C,D,I,J | No differences within sites | |
| *Caregiver physical activity behaviour^b^ (minutes)* | A,B,C,D,H,I,J | C,D,I,J | No differences within sites | |
| **Social Support** |  |  |  | |
| *Physical activity* | A,B,C,D,F,G,H,I,J | B,C,D,F,H,I,J | **Site A:**  Significant decrease pre-post, *t*(6) = 2.68, *p* = .04, *d* = .78, (Pre mean = 3.26 [SD: 0.88], post mean = 2.59 [SD: 0.99]) | |
| *Healthy eating* | A,B,C,D,H,I,J | B,C,D,I,J | No differences within sites | |
| **Healthy Related Quality of Life** | B,C,I,J | C,I,J | No difference within sites | |
|  | **Individual sites with sufficient data to conduct individual level analyses** | | |  |
| **Children** | **Pre-post** | **Pre-6-month** | **Findings** | |
| **Knowledge^a^** |  |  |  | |
| *Daily fruit and vegetable requirement* | A,B,C,E,F,G,H,I,J | B,C,D,E,H,J | No differences within sites | |
| *Sugary drinks (Module 3 only)* | A,D,E,F,H,J | B,D,H,J | No differences within sites | |
| *Daily physical activity* | All | B,C,D,E,H,I,J | No differences within sites | |
| *Screen time limitations* | All | B,C,D,E,H,I,J | No differences within sites | |
| **Efficacy** |  |  |  | |
| *Moderate-to-vigorous physical activity (0-100)* | A,B,C,D,E,F,G,I,J | B,C,D,I,J | **Site B:**  Significant difference between pre to 6mfu, *t*(2) = -5.05, *p* = .04, *d* = 2.89 (Pre mean = 56.11 [3.84], m6 = 68.33 [6.09] | |
| *Fruit and vegetable consumption^b^ (1-5)* | A,B,C,D,E,F,G,I,J | B,C,D,I,J | **Site C**  Significant difference from pre to post, p = .01, Z -2.55, *p = .*01, *r* = .85 (Pre Mdn = 2.75, Post Mdn = 3.5) | |
| *Eat breakfast^b^ (1-5)* | A,B,C,D,E,H | A,C,D,E | No differences within sites | |
| *Choose healthy drinks^b^ (1-5)* | A,B,C,D,E,H | A,C,D,E | No differences within sites | |
| *Cooking efficacy (1-5)* | A,B,C,D,E,F,H,J | B,C,D,I,J | No differences within sites | |
| **Behaviour** |  |  |  | |
| *Consumption of fruits and vegetables (1-7)* | A,B,C,E,F,I,J | B,C,H,J | No differences within sites | |
| *Consumption unhealthy food (1-7)* | A,B,C,E,F,H,I,J | B,C,H,J | **Site A:**  Significant increase from pre-post, *t*(8) = -2.32, *p* = .49, *d* = .12(Pre means =1.60 [.51], post mean = 2.16 [.93])  **Site H**  Significant decrease from pre – m6, *t*(7)=2.755, *p* = .03, *d =* 1.11 (Pre mean = 3.53 [.91], m6 = 2.51 [1.06])  **Site I**  Significant decrease from pre – post, *t*(3) = 4.38, *p* = .02, *d =*3.31 (Pre mean = 2.3 [.35], post mean = 1.4 [.20]) | |
| *Consumption of regular pop^b^ (1-7)* | A,B,C,E,F,H,I,J | B,C,H,I,J | **Site H:**  Significant difference from pre to post, Z = -2.07, p = .04, *r* = -.69, (Pre Mdn = 3.5, post Mdn = 3) | |
| *Eat breakfast^b^ (1-5)* | A,B,C,H,I,J | B,C,H,I,J | No differences within sites | |
| *Help cook^b^ (1-5)* | A,B,C,E,H,I,J | B,C,H,I,J | **Site J**  Significant difference from pre to post, Z = -2.27, *p* = .02, *r =* -.68(Pre Mdn = 3, post Mdn = 3) | |
| *Hours per day in front of screen (0-6)* | A,B,C,D,H,I,J | B,C,H,I,J | No differences within sites | |
| *Num of days spent in MVPA 30+mins* | A,B,C,E,H,I,J | B,C,H,I,J | No differences within sites | |
| **Social Support (0-4)** |  |  |  | |
| *Physical activity* | A,B,C,D,E,F,H,I,J | B,C,D,H,I,J | **Site A**  Significant increase from pre to post, *t*(12) = -2.28, *p* = .041, *d* = .35 (Pre mean = 2.61 [1.06], post mean = 3.03 [1.38]) | |
| *Healthy eating* | A,B,C,D,E,F,H,I,J | B,C,D,H,I,J | **Site A**  Sig increase from pre to post, *t*(12) = -2.28, *p*= .04, *d* = .3 (Pre mean = 3.28 [1.59], post mean = 3.71 [1.41] | |
| **Health Related Quality of Life (0-100)** | A,B,C,E,F,H,I,J | B,C,H,I,J | No differences within sites | |
