# Supplementary material for: Evaluation of a community-based, family focused healthy weights initiative using the RE-AIM framework
Source: Int J Behav Nutr Phys Act. 2018 Jan 26;15:13. doi: 10.1186/s12966-017-0638-0 (PMC5787319; doi:10.1186/s12966-017-0638-0)
Supplement: Supplementary file 5 — Statistics related to the Reach of the Healthy Together program. Description of data used examine program reach. (DOCX 14 kb) [file 12966_2017_638_MOESM5_ESM.docx]

| **Additional File 5.** Statistics related to the Reach of the Healthy Together program | | | | | | | |
| --- | --- | --- | --- | --- | --- | --- | --- |
| **Implementation Site** | | | **Inclusion or Exclusion Criteria**  (*Other than program age restrictions)* | **Population served by site if explicit** | | **Estimated number of families eligible for HT based on inclusion and exclusion criteria** | **Program Registration Numbers** |
| **Site A** | | No inclusion or exclusion criteria: “*We serve as many people that walk in the door”* | | | Focus on women and families | 5110 Families*  5255 Children 0-18* | Caregivers = 15  (*No caregivers Module 3)*  Children = 45 |
| **Site B** | | No inclusion or exclusion criteria: Recruitment for modules 2 and 3 originally targeted towards children in care but opened to any families with children in the target age group. | | | General population and children in care | 39030 Families*  33940 Children 0-18yrs* | Caregivers = 13  Children = 27 |
| **Site C** | | No inclusion or exclusion criteria: Recruitment originally targeted towards children in care but opened up to any families with children in the target age group. | | | Families of children from 0-6 years. | 5645 Families*  6490 Children 0-18yrs* | Caregivers = 85  Children = 100 |
| **Site D** | | Inclusion criteria: being from an immigrant or refugee families.  No exclusion criteria | | | Immigrant and refugee families. Newcomers to the country | 3403 Children 0-18 yrs* | Caregivers = 15  (*No caregivers Module 3)*  Children = 32 |
| **Site E** | | No inclusion or exclusion criteria | | | Primarily aboriginal families | 795 Families*  1575 Children 0-19yrs* | Caregivers = 14  Children = 29 |
| **Site F** | | No inclusion or exclusion criteria | | | Refugee and new immigrants to Canada | 15130 Families*  19140 Children 0-18yrs * | Caregivers = 18  Children = 22 |
| **Site G** | | No inclusion or exclusion criteria | | | Focus on aboriginal families | 1495 Families*  2130 Children 0-18yrs* | Caregivers = 4  (*No caregivers Module 3)*  Children = 13 |
| **Site H** | | Inclusion criteria: Recruitment targeted aboriginal and immigrant families within a specific school. Only families of children attending the school were eligible (***note***: families of children within that school who were not aboriginal or immigrants were not excluded from the program). | | | Work with high-risk families (aboriginal and families living in poverty) | Module 3: 600 students  Module 2: 435 students Module 1: 400 students (includes pre-K program though specific numbers not provided)+ | Caregivers = 22  (*No caregivers Module 3)*  Children = 62 |
| **Site I** | | No inclusion or exclusion criteria | | | Serve high-risk families (aboriginal and children in care) | **INFORMATION NOT AVAILABLE** | Caregivers = 12  (*No caregivers Module 3)*  Children = 22 |
| **Site J** | | No inclusion or exclusion criteria. Recruitment targeted individuals currently attended the center. | | | Vulnerable children | **INFORMATION NOT AVAILABLE** | Caregivers = 25  (*No caregivers Module 3)*  Children = 46 |
| * Based on census data of the service area specified by the organization  + Information obtained from community center director | | | | | | |  |
