# Supplementary material for: Evaluation of a community-based, family focused healthy weights initiative using the RE-AIM framework
Source: Int J Behav Nutr Phys Act. 2018 Jan 26;15:13. doi: 10.1186/s12966-017-0638-0 (PMC5787319; doi:10.1186/s12966-017-0638-0)
Supplement: Supplementary file 6 — Demographic characteristics of the caregivers at baseline based on site. (DOCX 21 kb) [file 12966_2017_638_MOESM6_ESM.docx]

| **Additional File 6.** Demographic characteristics of the caregivers at baseline based on site | | | | | | | | | | | |
| --- | --- | --- | --- | --- | --- | --- | --- | --- | --- | --- | --- |
| **Variable** | **Total**  **(n=126)** | **Site A**  **(n=14)** | **Site B**  **(n=12)** | **Site C**  **(n=18)** | **Site D**  **(n=17)** | **Site E**  **(n=0)** | **Site F**  **(n=13)** | **Site G**  **(n=4)** | **Site H**  **(n=17)** | **Site I**  **(n=11)** | **Site J**  **(n=20)** |
| **Age (y), mean (SD)** | 35.63 (10.80) | 35.85 (8.34) | 35.18 (16.52) | 37.17 (10.74) | 37.50 (5.09) |  | 39 (12.34) | 22.67 (19.09) | 31.67 (11.11) | 37.75 (4.57) | 37.40 (6.19) |
| **Gender (% female)** | 80.2 | 85.7 | 50 | 77.8 | 70.6 |  | 100 | 100 | 88.2 | 72.7 | 87.5 |
| **Relationship to Child in Program (%)** |  |  |  |  |  |  |  |  |  |  |  |
| Parent | 88.3 | 92.8 | 60 | 82.4 | 100 |  | 75 | 100 | 100 | 88.9 | 91.4 |
| Grandparent | 1.1 |  |  |  |  |  |  |  |  | 11.1 |  |
| Other adult related to child | 2.1 | 7.1 |  |  |  |  |  |  |  |  |  |
| Other adult not related to child | 8.5 |  | 40 | 17.6 |  |  | 25 |  |  |  | 5.9 |
| **Education (%)** |  |  |  |  |  |  |  |  |  |  |  |
| ≤ High school | 20.8 | 42.9 | 27.3 | 29.4 |  |  |  |  | 43.8 | 10 | 16.7 |
| High school or GED | 20.8 | 14.3 | 27.3 | 17.6 |  |  | 25 |  | 25 | 40 | 5.6 |
| Some College | 20.9 | 21.4 | 19 |  | 33.3 |  |  |  | 12.5 | 30 | 33.3 |
| College Degree | 27.1 | 21.4 | 27.3 | 35.3 | 33.3 |  | 50 | 66.7 | 12.5 | 20 | 27.8 |
| Graduate or professional degree | 10.4 |  |  | 17.6 | 33.3 |  | 25 | 33.3 | 6.3 |  | 16.7 |
| **Ethnicity of Caregiver (%)** |  |  |  |  |  |  |  |  |  |  |  |
| White | 42.5 | 14.3 | 81.8 | 93.3 |  |  | 33.3 | 66.7 | 9.1 | 50 | 18.8 |
| First Nations | 24.1 | 21.4 | 9.1 |  | 50 |  |  | 33.3 | 81.8 | 30 | 12.5 |
| Inuit | 4.6 | 28.6 |  |  |  |  |  |  |  |  |  |
| Metis | 3.4 | 7.1 |  |  |  |  |  |  | 9.1 | 10 |  |
| Indigenous | 2.3 | 7.1 |  |  |  |  |  |  |  |  | 6.3 |
| Chinese | 4.6 |  |  |  |  |  |  |  |  |  | 25 |
| South and Southeast Asian | 5.7 | 14.2 |  |  | 50 |  |  |  |  |  | 6.3 |
| Latin American | 2.3 |  |  |  |  |  |  |  |  |  | 12.5 |
| Filipino | 2.3 |  |  |  |  |  |  |  |  |  | 12.5 |
| Korean | 2.3 |  |  |  |  |  | 66.7 |  |  |  |  |
| Other | 5.9 | 7.1 | 9.1 | 6.7 |  |  |  |  |  | 10 | 6.3 |
| **Ethnicity of Child (%)** |  |  |  |  |  |  |  |  |  |  |  |
| White | 44.5 | 28.6 | 88.9 | 100 |  |  |  | 66.7 | 9.1 | 55.6 | 20 |
| First Nations | 24.7 | 14.3 | 11.1 |  | 50 |  |  | 33.3 | 81.8 | 33.3 | 13.3 |
| Inuit | 3.7 | 21.4 |  |  |  |  |  |  |  |  |  |
| Metis | 1.2 | 7.1 |  |  |  |  |  |  |  |  |  |
| Indigenous | 3.7 | 7.1 |  |  |  |  |  |  | 9.1 |  | 6.7 |
| Chinese | 4.9 |  |  |  |  |  |  |  |  |  | 26.7 |
| South and Southeast Asian | 3.7 |  |  |  | 50 |  |  |  |  |  |  |
| Latin American | 2.5 |  |  |  |  |  |  |  |  |  | 13.3 |
| Filipino | 2.5 |  |  |  |  |  |  |  |  |  | 13.3 |
| Korean | 2.5 |  |  |  |  |  | 66.7 |  |  |  |  |
| Other | 6.1 | 14.3 |  |  |  |  | 33.3 |  |  | 11.1 | 6.7 |
| **Children (%)** |  |  |  |  |  |  |  |  |  |  |  |
| 1 children in house | 31.4 |  |  |  |  |  |  |  |  |  |  |
| 2 children in house | 31.4 |  |  |  |  |  |  |  |  |  |  |
| ≥ 3 child in house | 37.3 |  |  |  |  |  |  |  |  |  |  |
| **Marital status n (%)** |  |  |  |  |  |  |  |  |  |  |  |
| Single | 20.8 | 21.4 | 36.4 | 29.4 |  |  |  | 33.3 | 20 | 10 | 16.7 |
| Married | 43.8 | 28.6 | 27.3 | 47.1 | 75 |  | 75 | 33.3 | 26.7 | 40 | 72.2 |
| Common-law | 28.1 | 50 | 36.4 | 23.5 |  |  |  | 33.3 | 53.3 | 30 |  |
| Separated/divorced | 5.2 |  |  |  | 25 |  | 25 |  |  | 20 |  |
| Widowed | 2.1 |  |  |  |  |  |  |  |  |  | 11.1 |
