# Supplementary material for: Evaluation of a community-based, family focused healthy weights initiative using the RE-AIM framework
Source: Int J Behav Nutr Phys Act. 2018 Jan 26;15:13. doi: 10.1186/s12966-017-0638-0 (PMC5787319; doi:10.1186/s12966-017-0638-0)
Supplement: Supplementary file 7 — Staff adoption of Healthy Together. Description of internal and external recruitment for Healthy Together across sites. (DOCX 54 kb) [file 12966_2017_638_MOESM7_ESM.docx]

| **Additional File 7**. Staff Adoption of Healthy Together | | | | |
| --- | --- | --- | --- | --- |
| **Implementation Site** | **Number of staff members employed at organization** | **Number of staff involved in HT (excluding director)** | **Number of staff hired from outside organization** | **Number of applicants** |
| **Site A** | 17 | 6 | 0 | 5 facilitators  2 program assistants |
| **Site B** | Information not provided | 9 | 0 | Individuals approached |
| **Site C** | 6 | 6 | 3 | 11 facilitators  2 program assistants |
| **Site D** | 123 | 7 | 0 | 3 facilitators  3 program assistants |
| **Site E** | 28 | 4 | 3 | 6 facilitators  8 program assistants |
| **Site F** | 31 | 7 | 0 | Only a few individuals interested (*specific numbers not provided)* |
| **Site G** | Information not provided | 8 | 0 | Information not provided |
| **Site H** | 30 | 9 | 0 | Individuals approached |
| **Site I** | 57 | *7* | 0 | 7 facilitators  5 program assistants |
| **Site J** | 28 | 9 | 1 | Many individuals interested (*specific numbers not provided)* |
