# Supplementary material for: Evaluation of a community-based, family focused healthy weights initiative using the RE-AIM framework
Source: Int J Behav Nutr Phys Act. 2018 Jan 26;15:13. doi: 10.1186/s12966-017-0638-0 (PMC5787319; doi:10.1186/s12966-017-0638-0)
Supplement: Supplementary file 8 — Healthy Together staff demographics. (DOCX 47 kb) [file 12966_2017_638_MOESM8_ESM.docx]

| **Additional File 8.** Healthy Together Staff Demographics | | | |
| --- | --- | --- | --- |
| **Variable** | **Coordinators**  ***(n =* 7)** | **Facilitators**  ***(n =* 27)** | **Program Assistants**  ***(n =* 20)** |
| **Age (y), mean (SD)** | 42±12 | 39(11) | 34±10 |
| **Education (%)** |  |  |  |
| High school |  | 1 (4%) |  |
| Apprenticeship or trades certificate or diploma |  | 1 (4%) | 1 (5%) |
| College, CEGEP or other non-university certificate or diploma | 1 (15%) | 3 (11%) | 4 (20%) |
| University certificate, diploma or degree | 6 (85%) | 17 (63%) | 13 (65%) |
| Post-graduate degree |  | 5 (18%) | 2 (10% |
| **Ethnicity of Caregiver (%)** |  |  |  |
| White | 3 (43%) | 17 (65%) | 14 (70%) |
| Native/Aboriginal | 2 (29%) | 2 (8%) | 2 (10%) |
| South and Southeast Asian |  | 3 (11%) |  |
| Latin American | 1 (14%) | 1 (4%) | 1 (5%) |
| Filipino |  | 2 (8%) |  |
| Black | 1 (14%) | 1 (4%) | 2 (10%) |
| Chinese |  |  | 1 (5%) |
