# Supplementary material for: Evaluation of a community-based, family focused healthy weights initiative using the RE-AIM framework
Source: Int J Behav Nutr Phys Act. 2018 Jan 26;15:13. doi: 10.1186/s12966-017-0638-0 (PMC5787319; doi:10.1186/s12966-017-0638-0)
Supplement: Supplementary file 9 — Facilitator demographics by site. (DOCX 15 kb) [file 12966_2017_638_MOESM9_ESM.docx]

| **Additional File 9.** Facilitator demographics by site | | | | | | | | | | |
| --- | --- | --- | --- | --- | --- | --- | --- | --- | --- | --- |
|  | **Site A**  **n = 1** | **Site B**  **n = 4** | **Site C**  **n = 2** | **Site D**  **n = 6** | **Site E**  **n = 2** | **Site F**  **n = 2** | **Site G**  **n = 1** | **Site H**  **n = 3** | **Site I**  **n = 3** | **Site J**  **n = 3** |
| ***Education*** | | | | | | | | | | |
| High school |  |  |  |  | 1 |  |  |  |  |  |
| Apprenticeship or trades certificate or diploma |  |  |  |  |  |  | 1 |  |  |  |
| College, CEGEP or other non-university certificate or diploma |  | 1 | 1, |  |  |  |  |  | 1 |  |
| University certificate, diploma or degree | 1 | 3 | 1 | 3 | 1 | 2 |  | 2 | 2 | 2 |
| Post-graduate degree |  |  |  | 3 |  |  |  | 1 |  | 1 |
| ***Ethnicity*** |  |  |  |  |  |  |  |  |  |  |
| White |  | 4 | 2 |  | 1 | 2 | 1 | 3 |  | 4 |
| Native/Aboriginal | 1 |  |  |  | 1 |  |  |  |  | 1 |
| South and Southeast Asian |  |  |  | 2 |  |  |  |  |  | 1 |
| Latin American |  |  |  | 1 |  |  |  |  |  |  |
| Filipino |  |  |  | 2 |  |  |  |  |  |  |
| Black |  |  |  | 1 |  |  |  |  |  |  |
| Chinese |  |  |  |  |  |  |  |  |  |  |
| ***Experience of working with children*** | | | | | | | | | | |
| Yes | 1 | 4 | 2 | 6 | 2 | 1 | 1 | 3 | 3 | 3 |
| No |  |  |  |  |  | 1 |  |  |  |  |
| ***Experience of working with children in care or at-risk children/youth*** | | | | | | | | | | |
| Yes | 1 | 4 | 2 | 3 | 2 | 1 |  | 2 | 3 | 3 |
| No |  |  |  | 3 |  |  | 1 | 1 |  |  |
