# Supplementary material for: Evaluation of a community-based, family focused healthy weights initiative using the RE-AIM framework
Source: Int J Behav Nutr Phys Act. 2018 Jan 26;15:13. doi: 10.1186/s12966-017-0638-0 (PMC5787319; doi:10.1186/s12966-017-0638-0)
Supplement: Supplementary file 10 — Evaluator’s Observations Module 1 (Data from 8 implementation sites). Description of specific module and session outcomes for Module 1 based on observations. (DOCX 16 kb) [file 12966_2017_638_MOESM10_ESM.docx]

| **Additional File 10.** Evaluator’s observations Module 1 (Data from 8 implementation sites) | | | | | | | |
| --- | --- | --- | --- | --- | --- | --- | --- |
| **Outcome** | **Session 1** | **Session 2** | **Session 3** | **Session 4*** | **Session 5** | **Comments** |  |
| % to which Session Objectives were met | 79  29-100 | 67  30-90 | 63  40-100 | 86  50-100 | 71  16.7-100 | Across all sites, no participants created a weekly meal plan for their family during session 2. In session 5 site J were largely unable to meet the program objectives, no explanation was provided. In session 1, site A felt they could not meet the program objectives as they ran out of time |  |
| % to which Activities/Resources were conducted as per protocol | 68 | 65 | 73 | 90 | 73 | No sites used the drinks video provided in session 2. Site D reported that the video did not work. Only sites D and H conducted the food label reading small group activity in session 2. In session 5, sites H, I and J did not complete the ‘Healthy Time Capsule’ activity, and site A sent home time capsule jars to conduct at home. |  |
| % of proposed discussions that were conducted | 63 | 78 | 39 | 63 | 81 | In session 1, sites A, B, I and J did not discuss what the participants want to get out of the program, what ‘Together’ means and sites A, D, H and J did not complete the “Family Meals’ discussion. In session 3, only sites B, C and G discussed Canada’s sedentary guidelines. In session 5, sites G, I and J did not discuss the benefits of family traditions. |  |
| % to which facilitators provided explanations as proposed | 65 | 79 | 68 | 52 | 54 | In session 5, only site D explained why monitoring a family’s physical activity is so important. |  |
| % to which the cooking activity was conducted | 100 | 100 | 100 | 100 | 100 | In session 5, site B prepared the ingredients for cooking before the session to save time. |  |
| % to which handouts were distributed | 59 | 71 | 70 | 66 | 81 | In session 2, site G did not distribute any handouts; however, the content of these handouts was discussed during the session. |  |
| Average Facilitators Preparation and Delivery Hours | 6.2 | 6.6 | 6.5 | 7 | 6.8 | In session 1, three facilitators (two from site C and one from site D) did not record hours spent in prep and delivery. In session 3, two facilitators from sites B and C did not record hours. In session 4, three facilitators (one each from sites E, H and J) did not record hours. |  |
| Average Program Assistants Preparation and Delivery Hours | 3.4 | 3.8 | 4.3 | 7.4 | 3.5 | In session 2, two program assistants from site B did not record hours spent in prep and delivery. In session 3, 2 program assistants (sites B and C) did not record hours. In session 4, 4 program assistants did not record hours (sites B, C, E and H). In session 5, three program assistants did not record hours (sites B, I and J). |  |
| *Data only from 7 implementation sites | | | | | | | |
