# Supplementary material for: Evaluation of a community-based, family focused healthy weights initiative using the RE-AIM framework
Source: Int J Behav Nutr Phys Act. 2018 Jan 26;15:13. doi: 10.1186/s12966-017-0638-0 (PMC5787319; doi:10.1186/s12966-017-0638-0)
Supplement: Supplementary file 11 — Evaluator’s Observations Module 2 (Data from 10 implementation sites). Description of specific module and session outcomes for Module 2 based on observations. (DOCX 16 kb) [file 12966_2017_638_MOESM11_ESM.docx]

| **Additional File 11.** Evaluator’s observations Module 2 (Data from 10 implementation sites) | | | | | | |
| --- | --- | --- | --- | --- | --- | --- |
| **Outcome** | **Session 1*** | **Session 2** | **Session 3** | **Session 4** | **Session 5** | **Comments** |
| % to which Session Objectives were met | 86  71-100 | 72  0-100 | 78  40-100 | 60  0-100 | 75  0-100 | One site evaluator did not provide feedback whether or not session objectives were met during session 2 (Site C), 4 (Site A) or 5 (Site J). In session 2, sites A, C, E, G and I discussed the role of culture in food. Sites D and E had participants who created a weekly meal plan for their family in session 2. |
| % to which Activities/Resources were conducted as per protocol | 74 | 70 | 87 | 83 | 70 | In session 1, site A did not do a physical activity and sites C and I used the Healthy Together PowerPoint presentation. In session 2 only site J used the drinks video. In session 3, site C, F, H, I and J did not give families time to set their SMART goals. Sites C, D, E and I completed the ‘Mapping Out Our Health’ activity and sites B, C, D, E and G conducted “Healthy Time Capsule’ activity in session 5. |
| % of proposed discussions that were conducted | 70 | 85 | 87 | 73 | 70 | Sites B, C, D, G and J discussed what ‘together’ means and conducted the “Family Meals’ discussion in session 1. Site B did not do discussion on “Family Traditions” in session 5 as the leaders felt that this would upset some children in care. |
| % to which facilitators provided explanations as proposed | 70 | 86 | 86 | 58 | 67 | In session 1, sites B, C, D and I provided an outline of the session ahead. |
| % to which the cooking activity was conducted | 100 | 90 | 100 | 100 | 100 | The site C evaluator did not provide answers regarding cooking for session 2. In session 3, site C did the cooking activity at the beginning of the session. |
| % to which handouts were distributed | 75 | 69 | 66 | 90 | 80 | Site B evaluator had the wrong observation feedback form and was not able to respond for 2 of the 3 handouts in session 4. Sites A, B, D, E and G handed out the ‘Cool Lunch Guide’ and ‘Dinner Talk Card Box’ handouts in session 2. |
| Average Facilitators Preparation and Delivery Hours | 9.1 | 6.7 | 5.8 | 5.3 | 5.8 | One site C facilitator reported spending 60 hours on prep and delivery in session 1. Two facilitators from site H and one from site J did not report hours for session 4. One facilitator from site C did not report hours for session 5. |
| Average Program Assistants Preparation and Delivery Hours | 3.5 | 3.5 | 2.8 | 3.7 | 2.6 | In session 2, one program assistant from site C did not record prep and delivery hours. In session 3, one program assistant from site H did not record hours. In session 4, two program assistants from sites C and H did not record hours. |
| *Data only from 8 implementation sites | | | | | | |
