# Supplementary material for: Evaluation of a community-based, family focused healthy weights initiative using the RE-AIM framework
Source: Int J Behav Nutr Phys Act. 2018 Jan 26;15:13. doi: 10.1186/s12966-017-0638-0 (PMC5787319; doi:10.1186/s12966-017-0638-0)
Supplement: Supplementary file 12 — Evaluator’s Observations Module 3 (Data from 10 implementation sites). Description of specific module and session outcomes for Module 3 based on observations. (DOCX 16 kb) [file 12966_2017_638_MOESM12_ESM.docx]

| **Additional File 12.** Evaluator’s observations Module 3 (Data from 10 implementation sites) | | | | | | |
| --- | --- | --- | --- | --- | --- | --- |
| **Outcome** | **Session 1** | **Session 2*** | **Session 3** | **Session 4** | **Session 5*** | **Comments** |
| % to which Session Objectives were met | 70  43-100 | 72  44.4-100 | 72  40-100 | 73  16.67-100 | 67  17-100 | One evaluator commented that she could not know how participants feel for specific objectives. Site A did not complete the majority of the session and went on to cooking portion in session 1. Site A felt that the program was too long for participants to maintain attention |
| % to which Activities/Resources were conducted as per protocol | 65 | 67 | 67 | 83 | 69 | In session one, sites A, J and H did not conduct physical activity. Sites C, D and I used the Healthy Together PowerPoint in session 1. No sites used the drinks video in session 2. In session 3, sites E, J and H did not complete a physical activity. Sites D, E and G provided time for participants to set SMART goals in session 3. In session 5, sites A, B, C, D and G completed the ‘Healthy Time Capsule’ activity. |
| % of proposed discussions that were conducted | 59 | 67 | 90 | 60 | 70 | In session 1, sites D, E and J discussed what ‘together’ means and sites B, C, D, F and G completed the ‘Family Meals’ discussion. In session 4, sites C, D, E, F and I conducted the discussions on ‘Physical Activity Recommendations’ and ‘Physical Activity that Reflects your Needs’. |
| % to which facilitators provided explanations as proposed | 69 | 79 | 68 | 65 | 56 | In session 1, sites D, E, H and I explained that participants might discover new things about themselves and their family. |
| % to which the cooking activity was conducted | 100 | 100 | 100 | 100 | 100 |  |
| % to which handouts were distributed | 67 | 60 | 63 | 75 | 74 | One site evaluator did not provide feedback on handouts in session 1 and 3. Only sites D and E handed out ‘Sip Smart BC!’ handout in session 2. |
| Average Facilitators Preparation and Delivery Hours | 10.2 | 5.2 | 6.2 | 5.4 | 5.4 | One site C facilitator reported spending 52 hours on prep and delivery of session 1. Three facilitators did not report hours for session 2. One facilitator did not hours for session 3. Two facilitators did no report hours for session 4. Two facilitators did no report hours for session 5. |
| Average Program Assistants Preparation and Delivery Hours | 4.3 | 5.6 | 5.5 | 5.3 | 5 | In session 2, one program assistant did not record prep and delivery hours. In session 3, one program assistant did not record hours. In session 4, one participant did not record hours. In session 5, one program assistant did not record hours. |
| *Data only from 9 implementation sites | | | | | | |
