# Supplementary material for: Evaluation of a community-based, family focused healthy weights initiative using the RE-AIM framework
Source: Int J Behav Nutr Phys Act. 2018 Jan 26;15:13. doi: 10.1186/s12966-017-0638-0 (PMC5787319; doi:10.1186/s12966-017-0638-0)
Supplement: Supplementary file 13 — Setting level maintenance of Healthy Together in the past year. (DOCX 15 kb) [file 12966_2017_638_MOESM13_ESM.docx]

| **Additional File 13**. Setting level maintenance of Healthy Together in the past year | | | | |
| --- | --- | --- | --- | --- |
| **Site** | **Was HT run in the past year?** | **Have aspects of HT been implemented in past year? If so, which ones** | **Reason** | **Source of information** |
| **Site A** | No | Utilized some HT material in existing program planning  (*Specific components not stated)* | No funding available to fully implement the program. Valued information provided within the program therefore incorporating into existing programs. | 1-year **email** follow-up Director |
| **Site B** | Yes  Five Times, 2 locations  One location taught two groups of teen parents and their children.  One location provided HT as an after-school program for 3 groups of children. | Plan to permanently integrate the activities and content of the HT into all existing programs.  (*Specific components not stated)* | Received a small amount of funding through an endowment grant within their agency to assist with the running of the program. Discussed having to be creative with activities and kitchen activities to ensure this small amount of money could cover program costs. | 1-year **email** follow-up Coordinator |
| **Site C** | No | No | Unable to run over the past year due to time constraints and a change in leadership at partner organization. Plan to run in future due to left over funds from initial implementation. Specifically, modules 2 and 3 in collaboration with local youth network. | 1-year follow-up **survey** Director and Coordinator |
| **Site D** | No | Incorporated aspects of HT into existing programming. Specifically, physical activity with preschoolers and discussion of screen time with parents. Conduct workshops on how to read food labels. Created a ‘Healthy snack’ aspect within other programs. | Program not run in full due to lack of funding. | 1-year follow-up **survey** Director and Coordinator |
| **Site E** | No | No | No reason provided | 1-year **email** follow-up Coordinator |
| **Site F** | No | Centre has made a conscious effort to offer healthy food/snacks within existing programs. | No reason provided | 1-year **email** follow-up Coordinator |
| **Site G** | No | Incorporated recipes into the existing community kitchen program. | Lack of funding to fully implement HT | 1-year **email** follow-up Coordinator |
| **Site H** | No | Incorporated aspects of HT into existing program within community (Fitness, Fun and Families).  Conscious about providing healthy snacks within existing programs.  Utilize HT handouts within other programs and recipes and physical activity with counseling clients. | Running the full program would not be possible without funding | 1-year follow up **survey** Coordinator |
| **Site I** | Yes  Module 2 conducted once (only first three sessions completed) | Used less of the suggested handouts and spent more time cooking, playing and connecting. | Stopped after 3-sessions due to lack of funding. No plans to run again due to lacks of funding. | 1-year follow-up **survey** Director and Coordinator |
| **Site J** | No | No | Lack of funding to fully implement HT and the program does not align well with the population this organization serves. | 1-year follow-up **survey** Director |
